# Prevalence of Hepatitis C Virus Infection in US States and the District of Columbia, 2013 to 2016

**DOI:** 10.1001/jamanetworkopen.2018.6371

**Published:** 2018-12-21

**Authors:** Eli S. Rosenberg, Elizabeth M. Rosenthal, Eric W. Hall, Laurie Barker, Megan G. Hofmeister, Patrick S. Sullivan, Patricia Dietz, Jonathan Mermin, A. Blythe Ryerson

**Affiliations:** 1 Department of Epidemiology and Biostatistics, University at Albany School of Public Health, State University of New York, Rensselaer; 2 Department of Epidemiology, Emory University Rollins School of Public Health, Atlanta, Georgia; 3 Division of Viral Hepatitis, National Center for HIV/AIDS, Viral Hepatitis, STD, and TB Prevention, Centers for Disease Control and Prevention, Atlanta, Georgia; 4 Office of the Director, National Center for HIV/AIDS, Viral Hepatitis, STD, and TB Prevention, Centers for Disease Control and Prevention, Atlanta, Georgia

## Abstract

**Importance:**

Infection with hepatitis C virus (HCV) is a major cause of morbidity and mortality in the United States, and incidence has increased rapidly in recent years, likely owing to increased injection drug use. Current estimates of prevalence at the state level are needed to guide prevention and care efforts but are not available through existing disease surveillance systems.

**Objective:**

To estimate the prevalence of current HCV infection among adults in each US state and the District of Columbia during the years 2013 to 2016.

**Design, Setting, and Participants:**

This survey study used a statistical model to allocate nationally representative HCV prevalence from the National Health and Nutrition Examination Survey (NHANES) according to the spatial demographics and distributions of HCV mortality and narcotic overdose mortality in all National Vital Statistics System death records from 1999 to 2016. Additional literature review and analyses estimated state-level HCV infections among populations not included in the National Health and Nutrition Examination Survey sampling frame.

**Exposures:**

State, accounting for birth cohort, biological sex, race/ethnicity, federal poverty level, and year.

**Main Outcomes and Measures:**

State-level prevalence estimates of current HCV RNA.

**Results:**

In this study, the estimated national prevalence of HCV from 2013 to 2016 was 0.84% (95% CI, 0.75%-0.96%) among adults in the noninstitutionalized US population represented in the NHANES sampling frame, corresponding to 2 035 100 (95% CI, 1 803 600-2 318 000) persons with current infection; accounting for populations not included in NHANES, there were 231 600 additional persons with HCV, adjusting prevalence to 0.93%. Nine states contained 51.9% of all persons living with HCV infection (California [318 900], Texas [202 500], Florida [151 000], New York [116 000], Pennsylvania [93 900], Ohio [89 600], Michigan [69 100], Tennessee [69 100], and North Carolina [66 400]); 5 of these states were in Appalachia. Jurisdiction-level median (range) HCV RNA prevalence was 0.88% (0.45%-2.34%). Of 13 states in the western United States, 10 were above this median. Three of 10 states with the highest HCV prevalence were in Appalachia.

**Conclusions and Relevance:**

Using extensive national survey and vital statistics data from an 18-year period, this study found higher prevalence of HCV in the West and Appalachian states for 2013 to 2016 compared with other areas. These estimates can guide state prevention and treatment efforts.

## Key Points

**Question:**

During 2013 to 2016, what proportion of adults were living with hepatitis C virus (HCV) infection in each US state?

**Findings:**

In this survey study, US national HCV prevalence during 2013 to 2016 was 0.93% and varied by jurisdiction between 0.45% and 2.34%. Three of the 10 states with the highest prevalence and 5 of the 9 states with the highest number of HCV infections were in the Appalachian region.

**Meaning:**

Regions with long-standing HCV epidemics, and those with newly emergent ones partly driven by the opioid crisis, face substantial HCV prevalence.

## Teaser

This survey study estimates the prevalence of current hepatitis C virus infection among adults in each US state and the District of Columbia from 2013 to 2016 using data from the National Health and Nutrition Examination Survey.

## Introduction

Hepatitis C virus (HCV) infection is the most frequently reported bloodborne infection in the United States and a leading cause of liver-related morbidity, transplantation, and mortality.^1^ Transmission of HCV occurs through exposure to infected body fluids, principally blood. Untreated, between 15% and 42% of infected persons resolve infection^2,3,4^; about half of those chronically infected develop progressive liver disease, which may include cirrhosis and hepatocellular carcinoma.^5,6^ Approximately 18 000 people died in 2016 because of HCV infection.^5,6,7^ Historically, HCV prevalence has been highest among persons in the birth cohort born between 1945 and 1965, and the number of people living with chronic infection was estimated to be 3.5 million in the late 2000s.^8,9^

Changes over the past decade have reshaped the US HCV epidemic. US Food and Drug Administration approval and increased availability of direct-acting antivirals have cured many people of infection.^10,11,12^ However, high all-cause and HCV-related mortality rates among persons in the highest-prevalence birth cohort for HCV infection remain.^13^ There has concomitantly been a tripling of HCV incidence, due primarily to an increase in persons injecting drugs and associated unsafe sharing of injection equipment related to the opioid crisis.^7,14,15^

With the increasing availability of direct-acting antivirals, national and state-level public health strategies have raised elimination of HCV as a possible goal. Accurate estimates of the current burden of HCV infection in each US jurisdiction are critical to the policy, programmatic, and resource planning of elimination strategies. However, national case surveillance provides an incomplete picture of the burden of HCV infection. Although HCV infection is reportable to the Centers for Disease Control and Prevention’s (CDC’s) National Notifiable Diseases Surveillance System, acute and chronic infections reported through this program represent a small proportion of cases, and in some states neither are reportable.^7,16^ Some jurisdictions maintain enhanced surveillance programs funded by the CDC or other sources, yet a comprehensive jurisdiction-specific picture for the nation remains inestimable from case surveillance data. The current approach for estimating national HCV prevalence involves analysis of the US National Health and Nutrition Examination Survey (NHANES), which conducts HCV testing among noninstitutionalized persons aged 6 years or older.^8,17^ An updated national HCV prevalence for 2013 to 2016 has been estimated using NHANES, reflecting the previously mentioned age-bimodal epidemic patterns, yielding an estimated 2.4 million persons with HCV RNA–positive results, indicating current (acute or chronic) infection.^12^ This estimate used methods to account for populations unrepresented in NHANES-based estimates, including individuals experiencing incarceration and unsheltered homelessness, groups that represent 11% of HCV prevalence. Current subnational estimates are needed to guide local HCV elimination efforts, as previous estimates are no longer valid owing to changes in HCV epidemiology over the past few years.

We present an updated approach to our previous methodology for state-specific HCV prevalence estimation that reflects current changes to the epidemic.^18^ This method uses newly released NHANES and vital statistics data through 2016 and incorporates HCV-related and narcotic overdose deaths to yield updated estimates that reflect overlaid spatial patterns in HCV infection attributable to previous and recent transmission.

## Methods

We used a multistep statistical approach (eFigure 1 in the Supplement), first generating direct estimates for each state using NHANES national prevalence in sex, race/ethnicity, birth cohort, and poverty strata. We next examined the distribution of each state’s cause-specific death rates relative to the US average as signals for local patterns of HCV infection. Within demographic strata, we applied 2 sets of state-specific mortality ratios relative to the nation, mortality rates from HCV infection and narcotic overdose, to represent older and recent infections, respectively. We then estimated additional infections among populations not included in NHANES’ sampling frame by applying literature-based estimates of prevalence in these groups to state-specific population estimates. All analyses were limited to persons aged 18 years or older. In the following section, we describe this approach in detail. This study was reported according to the American Association for Public Opinion Research (AAPOR) reporting guideline. Because the study used publicly available data, institutional review board approval was not sought per organizational policy.

### Data Sources

#### NHANES (1999-2016)

Every 2-year cycle, NHANES samples approximately 10 000 individuals through a complex multistage design that represents the noninstitutionalized civilian US population.^17^ The survey collects demographic characteristics and specimens for HCV RNA and antibody testing.^19,20^ Additional details, including response rates, are in eAppendix 1 in the Supplement.

Race/ethnicity was categorized into non-Hispanic black and other race/ethnicities. Birth year was categorized as before 1945, 1945 to 1969, and after 1969. The typical 1945 to 1965 birth cohort with the highest HCV prevalence was expanded by 4 years because preliminary NHANES analyses showed similar prevalence to the traditional birth cohort (not shown). Income was represented as a ratio comparing family income with the US Department of Health and Human Services poverty guidelines for each year and categorized in the following groups: below the federal poverty level, 1.0 to 1.9 times the federal poverty level, and 2.0 times the federal poverty level or more.^8^ Missing income data (n = 3931 [8.30%]) were imputed using a process described in eAppendix 2 in the Supplement. We pooled 9 data cycles (1999-2016) to ensure sufficient stratum-level data (Table 1).

**Table 1.** Data Sources

Data Source	Years Included	Purpose	Individuals Represented, No.	Cases, No.	Data Extraction Notes
NHANES	1999-2016	National HCV RNA prevalence overall and by strata of sex, race/ethnicity, birth cohort, and poverty; trends in HCV antibody inform analysis weights	47 387 With nonmissing HCV RNA test results; 47 590 with nonmissing HCV antibody test results^a^	575 With positive HCV RNA test; 874 with positive HCV antibody test^a^	NHANES 2000, 2002, 2004, 2006, 2008, 2010, 2012, 2014, 2016 data sets
US Census intercensal data	1999-2016	Population structure for modeling HCV- and overdose-related mortality rates	4 109 869 228 Person-years aged ≥18 y	NA	US Vintage 2000, Vintage 2009, Vintage 2016 data sets
US Census American Community Survey	2012-2016	Noninstitutionalized US population structure for final estimates	12 023 450 Observations of noninstitutionalized persons aged ≥18 y	NA	5-y Public Use Microdata Sample
National Vital Statistics System	1999-2016	Distribution of hepatitis C–related mortality, signaling underlying HCV prevalence, to inform distribution of older HCV infections	44 071 310 Decedents aged ≥18 y who resided in the 50 states or the District of Columbia	261 858 With HCV as underlying or multiple cause of death	*ICD-10* codes included acute viral hepatitis C (B17.1) and chronic viral hepatitis C (B18.2)
National Vital Statistics System	1999-2016	Distribution of narcotic overdose mortality, signaling underlying injection patterns, to inform distribution of newer HCV infections	44 071 310 Decedents aged ≥18 y who resided in the 50 states or the District of Columbia	541 130 With unintentional or undetermined cause narcotic or unknown drug as underlying or multiple cause of death	*ICD-10* codes included poisoning by and exposure to narcotics and psychodysleptics (hallucinogens) (X42 unintentional, Y12 undetermined intent); poisoning by and exposure to other and unspecified drugs, medicaments, and biological substances (X44 unintentional, Y14 undetermined intent)

Abbreviations: HCV, hepatitis C virus; *ICD-10*, *International Classification of Diseases, Tenth Revision*; NA, not applicable; NHANES, National Health and Nutrition Examination Survey.

^a^ Hepatitis C virus antibody screening test data are included for all years. Confirmatory test data for HCV antibodies are not publicly available for 2015 to 2016.

#### American Community Survey Public Use Microdata Sample (2012-2016)

The American Community Survey (ACS) samples nearly 3 million addresses annually, collecting demographic and economic characteristics of the US population.^21^ We used the 2012 to 2016 five-year ACS Public Use Microdata Sample^22^ to estimate population denominators for the noninstitutionalized population in each stratum and state. Race/ethnicity, birth year, and income were categorized as we have described, and we conducted imputation analyses for missing income data (n = 237 600 [1.98%]) (eAppendix 2 in the Supplement).

#### National Vital Statistics System Multiple Cause of Death Mortality Data (1999-2016)

Multiple Cause of Death Mortality Microdata files (1999-2016), including individual death records for persons who lived in a US state or the District of Columbia, were requested from the National Vital Statistics System (NVSS).^23^ These records contained *International Classification of Diseases, Tenth Revision* (*ICD-10*) codes for multiple underlying causes of deaths (N = 44 071 310).

Hepatitis C virus–related mortality was classified using the *ICD-10* code for acute viral hepatitis C (B17.1) or chronic viral hepatitis C (B18.2) as an underlying or multiple cause of death (n = 261 858). We earlier demonstrated that although HCV is underreported on death certificates, HCV prevalence estimates were not meaningfully affected because underreporting was insufficiently differential by jurisdiction.^18^

Narcotic overdose mortality, an outcome highly correlated with local acute HCV infection,^24^ was classified using the *ICD-10* codes for unintentional poisoning by and exposure to narcotics and psychodysleptics (hallucinogens) (X42), unknown intention poisoning by and exposure to narcotics and psychodysleptics (hallucinogens) (Y12), unintentional poisoning by and exposure to other and unspecified drugs, medicaments, and biological substances (X44), or unknown intention poisoning by and exposure to other and unspecified drugs, medicaments, and biological substances (Y14) (n = 541 130). This algorithm is more specific to injection-related overdose deaths than others, while robust to missingness, with full considerations described in eAppendix 3, eFigure 2, and eTables 1 and 2 in the Supplement.^25,26,27^

### Analysis

#### NHANES-Eligible Population

The equation in eAppendix 4 in the Supplement details our estimator for the total persons with HCV in each state in the NHANES population, depicted visually in eFigure 1 in the Supplement. Within 12 strata representing previously defined levels of sex, race/ethnicity, and birth year, we computed the standardized estimate by direct estimation from a weighted logistic regression model of NHANES, which included the terms for these strata, era (1999-2012 and 2013-2016), and poverty. To yield standardized estimates for the 12 demographic strata that accounted for poverty, we output logistic model estimates for the 2013 to 2016 era and weighted them according to the ACS poverty distribution for the 12 strata in each state.

Next, we estimated the state stratum-specific likelihood of HCV-related mortality, using a logistic model of NVSS-derived mortality counts, per person-years, that approximated full-stratification with main effects for state, sex, race/ethnicity, birth cohort, era; 2-way interactions for state by each sex, race/ethnicity, birth cohort, and era; 2-way and 3-way interactions for each combination of sex, race/ethnicity, birth cohort, and era; and 4-way interaction of sex, race/ethnicity, birth cohort, and era. These state stratum-specific mortality estimates were divided by the national stratum-specific average, yielding a mortality ratio for the state stratum. This process was repeated for the narcotic overdose mortality. The 2 mortality ratios per stratum were averaged according to weights *w_j_* (values described in the following section) and then multiplied by the standardization-based value to yield adjusted totals. Summing these across all 12 state strata yielded the estimated number of persons with HCV, which when divided by the ACS state population *N_i_* yielded the estimated prevalence rate.

#### Weights

In the primary analysis, 3 weights *w_j_* were used, with the same *w_j_* applied to the 4 sex–race/ethnicity strata within birth cohort, representing the proportion of that birth cohort’s current infections allocated as prevalent in 1999 to 2012 (*w_j_*) vs incident during 2013 to 2016 (1 − *w_j_*). For persons born before 1945, we assumed no recent infections due to injection (*w_j_* = 1). Based on additional analyses of biannual NHANES trends in HCV-antibody and literature estimates, we set *w_j_* = 0.875 for those born from 1945 to 1969, and *w_j_* = 0.378 for those born after 1969 (eAppendix 5 and eTables 3 and 4 in the Supplement). To facilitate comparisons with our earlier approach for 2010, which considered only HCV mortality, we conducted a sensitivity analysis with all *w_j_* = 100%.^18^ An additional sensitivity analysis considered an upper bound for incidence among persons born from 1945 to 1969, with *w_j_* = 0.80 (eAppendix 5 and eTables 3 and 5 in the Supplement).

#### Confidence Intervals

Confidence intervals accounted for the joint statistical uncertainty from the 3 logistic regression models and 2 poverty imputation models. This was done with a Monte Carlo simulation that resampled parameter estimates from logit-normal distributions, using the standard errors for each, and recomputed all modeling steps (*k* = 10 000 runs) to produce 95% CIs.

### Additional Populations

The National Health and Nutrition Examination Survey does not sample persons who are incarcerated, experiencing unsheltered homelessness, or residing in nursing homes. We expanded to states the earlier-described method for including these populations nationally.^12^ In brief, for incarcerated and homeless populations, HCV prevalence was estimated based on values identified in a systematic literature review of articles published from January 1, 2013, to December 31, 2017. For incarcerated populations, the mean prevalence of the literature estimates was generated using a random-effects model with study sample size as weight. For nursing home residents, the age-sex standardized NHANES prevalence was used. State-level population size estimates for these groups as of December 31, 2016, were obtained from public data sources. Additional detail on data sources and prevalence estimates appears in eTable 6 in the Supplement. For each state, within each population, we multiplied the national HCV prevalence rate by the state-specific population size to yield the number infected, which was then summed across populations to yield the state total persons with HCV among additional populations. We also conducted a secondary analysis that further adjusted by state-specific prevalence rates in the NHANES-represented population (eAppendix 6 in the Supplement).

State-level estimates for populations not represented in NHANES were added to the model results within each state, allowing calculation of point estimates for prevalence in the total state population.

Unlike the national analysis,^12^ we did not account for active-duty military populations in our state approach because this group consists of persons originating from multiple states residing in facilities outside of state jurisdiction, for whom data on origin states are unavailable and for whom there exists insufficient evidence of increased HCV risk. This population represents an estimated 6900 persons with HCV infection in the United States (0.3%).^12^

## Results

For the years 2013 to 2016, we estimated an HCV RNA prevalence of 0.84% (95% CI, 0.75%-0.96%) among adults in the noninstitutionalized US population represented in the NHANES sampling frame, corresponding to 2 035 100 (95% CI, 1 803 600-2 318 000) persons with current infection (Table 2). Accounting for populations not included in NHANES, there were 231 600 additional persons with HCV, adjusting prevalence to 0.93% (10% relative increase nationally with a state increase range of 2%-23%), with prevalence relatively increasing by more than 20% in Georgia and South Dakota and less than 5% in Rhode Island and the District of Columbia. These deviations were largely attributable to respectively higher and lower proportions of persons incarcerated in these jurisdictions (data not shown). Using the alternative method that adjusted additional populations for background state prevalence in the NHANES population, the relative proportional change from the primary method was minimal (state median [range] change, −0.5% [−7.6% to 8.4%]) (eTable 7 in the Supplement).

**Table 2.** Estimated Total and Prevalence of Persons With Current HCV Infection, US States and District of Columbia, 2013 to 2016

State	2016 Adult Population, No.^a^	Population Included in NHANES Sampling Frame		With Additional Populations Not Included in NHANES Sampling Frame	
		HCV RNA Positive (95% CI), No.^b^	% (95% CI)^c^	HCV RNA Positive, No.^b^	Total Adult Population 2016, No. (%)
Alabama	3 671 100	26 100 (23 100-29 600)	0.71 (0.63-0.81)	30 700	3 736 700 (0.82)
Alaska	542 500	4700 (3900-5700)	0.86 (0.72-1.05)	5200	548 000 (0.95)
Arizona	5 020 500	55 300 (48 000-64 100)	1.10 (0.96-1.28)	61 500	5 090 500 (1.21)
Arkansas	2 215 500	19 100 (16 800-21 800)	0.86 (0.76-0.99)	21 800	2 258 700 (0.97)
California	29 160 200	288 500 (253 500-331 800)	0.99 (0.87-1.14)	318 900	29 544 700 (1.08)
Colorado	4 057 000	32 500 (28 000-38 400)	0.80 (0.69-0.95)	36 300	4 108 500 (0.88)
Connecticut	2 771 800	16 500 (14 200-19 700)	0.60 (0.51-0.71)	18 300	2 812 700 (0.65)
Delaware	719 400	5600 (4800-6500)	0.78 (0.67-0.90)	6300	730 500 (0.86)
District of Columbia	537 500	12 400 (10 500-14 800)	2.32 (1.95-2.76)	12 700	542 400 (2.34)
Florida	15 620 600	133 200 (117 700-152 100)	0.85 (0.75-0.97)	151 000	15 860 200 (0.95)
Georgia	7 465 900	46 400 (41 300-52 300)	0.62 (0.55-0.70)	56 800	7 597 700 (0.75)
Hawaii	1 094 200	5700 (4700-7000)	0.52 (0.43-0.64)	6700	1 107 400 (0.60)
Idaho	1 187 300	9900 (8400-11 800)	0.84 (0.71-0.99)	11 200	1 203 300 (0.93)
Illinois	9 703 700	47 700 (42 200-54 300)	0.49 (0.44-0.56)	54 900	9 842 400 (0.56)
Indiana	4 915 800	35 400 (30 900-40 700)	0.72 (0.63-0.83)	40 200	5 000 100 (0.80)
Iowa	2 339 900	11 100 (9 500-13 100)	0.47 (0.40-0.56)	12 600	2 379 300 (0.53)
Kansas	2 137 000	12 600 (10 900-14 800)	0.59 (0.51-0.69)	14 600	2 173 600 (0.67)
Kentucky	3 331 500	38 600 (33 600-44 800)	1.16 (1.01-1.34)	42 500	3 390 700 (1.25)
Louisiana	3 445 000	44 900 (40 000-50 400)	1.30 (1.16-1.46)	50 000	3 518 500 (1.42)
Maine	1 058 600	6500 (5400-7800)	0.61 (0.51-0.74)	7000	1 069 400 (0.65)
Maryland	4 547 800	37 300 (32 700-43 100)	0.82 (0.72-0.95)	40 600	4 602 900 (0.88)
Massachusetts	5 283 400	35 800 (30 600-42 500)	0.68 (0.58-0.80)	38 100	5 346 600 (0.71)
Michigan	7 578 400	62 800 (55 800-70 900)	0.83 (0.74-0.94)	69 100	7 676 600 (0.90)
Minnesota	4 115 000	22 300 (19 400-26 000)	0.54 (0.47-0.63)	24 300	4 159 900 (0.58)
Mississippi	2 205 500	19 600 (17 500-22 200)	0.89 (0.79-1.01)	22 900	2 251 700 (1.02)
Missouri	4 575 700	35 200 (31 100-40 200)	0.77 (0.68-0.88)	40 300	4 660 800 (0.86)
Montana	787 100	6800 (5700-8000)	0.86 (0.73-1.02)	7400	798 100 (0.93)
Nebraska	1 391 400	6900 (6000-8200)	0.50 (0.43-0.59)	7900	1 412 800 (0.56)
Nevada	2 148 500	19 300 (16 800-22 400)	0.90 (0.78-1.04)	21 900	2 177 400 (1.00)
New Hampshire	1 046 300	7200 (5900-8900)	0.69 (0.57-0.85)	7700	1 058 000 (0.73)
New Jersey	6 810 300	43 400 (37 900-50 300)	0.64 (0.56-0.74)	47 200	6 890 900 (0.68)
New Mexico	1 557 100	25 000 (21 600-29 100)	1.61 (1.39-1.87)	26 700	1 578 000 (1.69)
New York	15 260 100	107 100 (94 900-121 600)	0.70 (0.62-0.80)	116 000	15 448 400 (0.75)
North Carolina	7 545 400	60 200 (53 600-68 100)	0.80 (0.71-0.90)	66 400	7 640 100 (0.87)
North Dakota	559 100	2200 (1800-2800)	0.39 (0.32-0.50)	2600	568 300 (0.45)
Ohio	8 787 100	81 500 (71 800-93 200)	0.93 (0.82-1.06)	89 600	8 938 500 (1.00)
Oklahoma	2 862 800	48 900 (42 700-56 500)	1.71 (1.49-1.97)	53 300	2 922 700 (1.82)
Oregon	3 086 200	45 700 (39 400-53 700)	1.48 (1.28-1.74)	48 700	3 120 900 (1.56)
Pennsylvania	9 888 700	84 500 (74 300-97 000)	0.86 (0.75-0.98)	93 900	10 055 600 (0.93)
Rhode Island	829 900	9600 (8300-11 400)	1.16 (1.00-1.37)	10 000	841 300 (1.19)
South Carolina	3 689 100	31 900 (28 400-36 100)	0.87 (0.77-0.98)	35 600	3 740 300 (0.95)
South Dakota	628 400	3000 (2500-3700)	0.48 (0.39-0.59)	3700	641 000 (0.57)
Tennessee	4 972 200	63 500 (56 200-72 100)	1.28 (1.13-1.45)	69 100	5 053 700 (1.37)
Texas	19 455 200	178 000 (157 500-203 100)	0.91 (0.81-1.04)	202 500	19 777 300 (1.02)
Utah	2 024 600	11 000 (9300-13 100)	0.54 (0.46-0.65)	12 300	2 042 200 (0.60)
Vermont	499 100	3500 (2900-4200)	0.70 (0.58-0.85)	3700	503 800 (0.73)
Virginia	6 348 500	33 500 (29 400-38 500)	0.53 (0.46-0.61)	39 900	6 436 400 (0.62)
Washington	5 412 700	50 000 (43 100-58 900)	0.92 (0.80-1.09)	54 200	5 468 900 (0.99)
West Virginia	1 439 300	19 500 (16 700-23 000)	1.35 (1.16-1.60)	20 600	1 459 400 (1.41)
Wisconsin	4 384 900	24 000 (21 000-27 700)	0.55 (0.48-0.63)	27 900	4 449 600 (0.63)
Wyoming	437 600	3200 (2600-3900)	0.73 (0.60-0.90)	3700	444 300 (0.82)
Total^d^^,^^e^	241 152 600	2 035 100 (1 803 600-2 318 000)	0.84 (0.75-0.96)	2 266 700	244 681 600 (0.93)^f^

Abbreviations: HCV, hepatitis C virus; NHANES, National Health and Nutrition Examination Survey.

^a^ Population sizes are estimated as of December 2016 based on American Community Survey 5-year estimates from 2012 to 2016 and include noninstitutionalized adults eligible for NHANES. This estimate includes 1 288 600 active-duty military personnel ineligible for NHANES, which cannot be removed at the state level because population sizes are unavailable by home state of personnel. Therefore, this assumes a mean prevalence value for this group, adding 5000 infections nationally.

^b^ Number of infected persons is calculated by multiplying the prevalence percentage estimate by the adult population size before rounding for presentation.

^c^ The NHANES prevalence percentage estimates are based on results from 2013 to 2016 NHANES. Population size includes noninstitutionalized adults eligible for NHANES from the 2012 to 2016 American Community Survey.

^d^ Values may not sum to total due to rounding.

^e^ Results are based on a regression model that incorporates data for the period 1999 to 2016 and generates estimates via simulations. Accordingly, these results do not precisely sum to previous national totals for the 2013 to 2016 period.^11^

^f^ Does not sum to previous 2013 to 2016 US total due to the exclusion of persons incarcerated in federal prisons who are not assigned to state-specific populations.^11^

**Figure 1.**
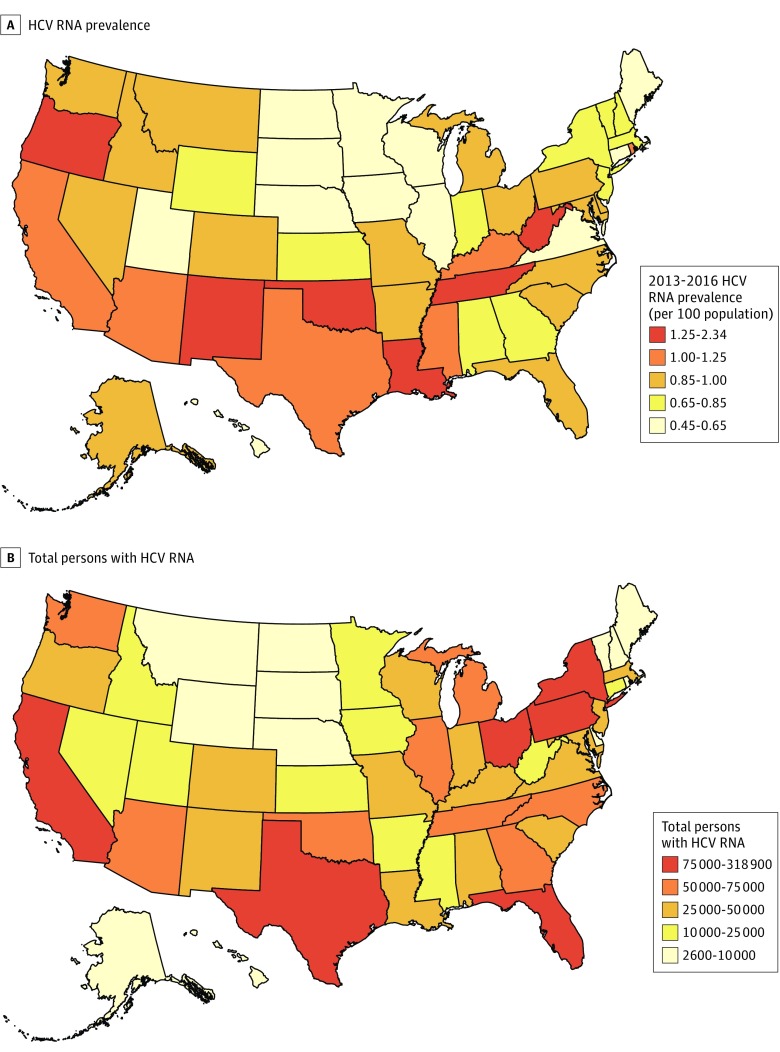
Estimated Hepatitis C Virus (HCV) RNA Prevalence and Total Persons With HCV RNA, Indicating Current Infection, United States and District of Columbia, 2013 to 2016 Prevalence of HCV (A) and total number of persons with HCV (B) in the full US adult population defined by noninstitutionalized adults included in the National Health and Nutrition Examination Survey sampling frame and additional populations not in the sampling frame (those incarcerated, in nursing homes, and experiencing homelessness).

Large variations were observed in total population HCV prevalence by state (median [range], 0.88% [0.45%-2.34%]) (Figure 1). Of 13 states in the US West census region, 10 were above this median rate, and the region contained 27.1% of infected persons, despite constituting 23.4% of the US population. Three of the 10 states with the highest rates are members of the US Appalachian Regional Commission (Kentucky, Tennessee, and West Virginia) and together constituted 5.8% of persons with HCV and 4.0% of the population.^28^ Nine states (California [318 900], Texas [202 500], Florida [151 000], New York [116 000], Pennsylvania [93 900], Ohio [89 600], Michigan [69 100], Tennessee [69 100], and North Carolina [66 400] each contained more than 65 000 persons with HCV and together constituted 51.9% of all persons with HCV nationally. Of these 9 states, 5 are in the Appalachian region (New York, North Carolina, Ohio, Pennsylvania, and Tennessee). Tennessee and Arizona were the only states represented in the top 10 for both HCV rates and persons with HCV.

**Figure 2.**
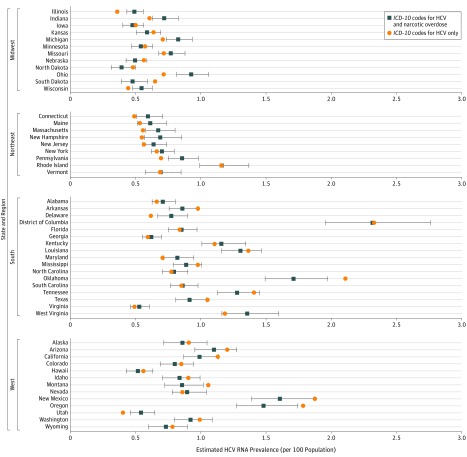
Hepatitis C Virus (HCV) RNA Prevalence, Accounting for the Distribution of Both HCV and Narcotic Overdose Deaths or HCV Deaths Only, by US State and Census Region, 2013 to 2016 Prevalence in the US adult population defined by noninstitutionalized adults included in the National Health and Nutrition Examination Survey sampling frame. Error bars represent 95% confidence intervals. ICD-10 indicates *International Classification of Diseases, Tenth Revision*.

Figure 2 displays the impact of the revised methodology for the NHANES population that incorporates the distribution of narcotic overdose mortality, relative to considering HCV mortality only. States experiencing higher rates of overdose mortality saw relative increases in estimated HCV prevalence, whereas those with lower rates saw declines in prevalence. A sensitivity analysis that considered maximally increased weighting of overdose mortality (and incidence) in the 1945 to 1969 birth cohort yielded small proportional changes from the default weighting (median [range], 0.3% [−4.8% to 5.7%]) (eTable 5 in the Supplement).

## Discussion

Using newly available data for 2013 to 2016 and methods that account for changes in HCV epidemiology, we observed large variation in HCV prevalence and burden across the United States. There was a particularly high prevalence in the West and Appalachia. These findings were consistent across analyses that considered alternative incidence rates for the highest-prevalence 1945 to 1969 cohort and alternative approaches for populations not included in the NHANES sampling frame.

The state patterns for areas of high burden, particularly the Appalachian region, closely echo recent reports of direct, local (but incomplete) measures of HCV burden using acute HCV surveillance in the National Notifiable Diseases Surveillance System and maternal HCV status on birth certificates in NVSS.^29,30^ In Appalachia, it is likely that HCV prevalence reflects recent increases in injection drug use, high densities of counties vulnerable to HCV and HIV infection outbreaks, large outbreaks of these infections among persons who inject drugs (PWID), and elevated reports of acute HCV.^7,14,24,31^

These estimates help to quantify the need for investments in efficacious direct and indirect services for the prevention of HCV acquisition and transmission. This includes syringe services programs, which are associated with decreased HCV spread, especially when combined with linkage to medication-assisted substance use treatment.^32^ Although increasing, the number of syringe services programs remains low in 2018 in many states, with programs often geographically dispersed within states.^33^ Direct medical services such as HCV testing and curative treatments remain cornerstones for extending life and averting transmission.^34^ Furthermore, testing and treatment are cost-effective, with earlier treatment possibly yielding greater cost savings.^35,36,37^

Despite availability of these services, some policies restrict their use. A recent analysis found substantial variation in the comprehensiveness of laws supporting access to clean injection equipment and sobriety requirement–based restrictions of Medicaid fee-for-service HCV treatment.^29^ Some of the highest-incidence states had the lowest levels of prevention and treatment access overall, with 47 states lacking comprehensive laws and Medicaid policies for effective prevention and treatment of HCV among PWID. Additionally, restrictions based on fibrosis score remain prevalent, and a 45-state analysis of 2016 to 2017 pharmacy data found treatment had been denied for many patients with Medicaid (34.5%) and private insurance (52.4%).^38^ Finally, indirect HCV prevention is achievable by addressing opioid use disorder using efficacious individual approaches, like medication-assisted treatment, and numerous state- and systems-level policies.^39,40^

Even with effective tools for addressing the HCV epidemic, substantial challenges remain in their application to rural PWID. The evidence base for understanding the unique HCV risk, prevention, and care context of these areas remains limited.^41^ Others have prioritized areas for further research and recent federal commitments are promising.^42,43^

### Limitations

Key strengths of our approach include anchoring to robust and comprehensive national data systems, use of highly specific markers of local HCV infection that reflect the bimodal epidemic pattern, and a near-exact standardization approach, yet several limitations remain. It is possible that HCV increases associated with PWID are not well represented in national NHANES estimates. However, earlier analyses demonstrated robustness for this subgroup,^12^ lifetime exposure among those born in 1970 or later (per eTable 4 in the Supplement) indicate dramatic increases consistent with acute surveillance trends, and estimated national totals are consistent with projections from a population-based dynamic model.^37^ A recently published analysis of laboratory databases reports a number of persons diagnosed that exceeds previous national prevalence estimates, likely due to incomplete deduplication of infections across deidentified databases.^44^ Mortality caused by HCV may be an imperfect spatial marker given underreporting, although we previously demonstrated that the method is robust to this.^13,18^ Likewise, limitations may exist with the use of narcotic overdoses to represent recent infection. First, further specificity for likely injected narcotic, per toxicology codes, remains challenging because of substantial data toxicology code missingness.^27^ Additionally, to the extent that more lethal narcotics such as fentanyl are more prevalent in certain jurisdictions, this may bias estimates upward. Further refinements of toxicology code data are required to account for this. Second, local variations may exist in the relationship between overdose deaths and HCV-risky injection. State-specific variations in laws and funding of interventions that avert overdose deaths, like naloxone, and those that reduce HCV risks associated with injection while influencing mortality less, like syringe services programs, may bias estimates in opposing directions.^33,39,45^ Estimates for populations not included in NHANES are based on systematic approaches, but still may not be representative. Explorations of the impact of variations in these estimates found this contributed little overall variation. Ultimately, one of the best ways to overcome these limitations, particularly as jurisdictions wish to monitor progress in shorter time frames, is to strengthen core surveillance registries through standardized reporting definitions, active case finding, and rigorous linkages to understand mortality, treatment, and migration.^46,47,48^ Third, our state estimate sum is slightly lower than the recent updated national estimate, owing to 2 methodological differences: use of a weighted regression model that pools a broader time period and noninclusion of active-duty military persons.^12^

## Conclusions

Prevalence of HCV infection varies widely in the United States. Highest rates are frequently in states deeply affected by the opioid crisis or with a history of increased levels of injection drug use and chronic HCV infection, particularly in the West. Progress toward hepatitis C elimination is theoretically possible with the right investments in prevention, diagnosis, and cure.^34,47,49,50^ The urgency for action and the resources necessary will vary by jurisdiction.

## Notes

Supplement. **eAppendix 1.** NHANES Methodological Details **eAppendix 2.** Imputation for Missing Poverty Data in the National Health and Nutrition Examination Survey (NHANES) and the American Community Survey (ACS) **eAppendix 3.** Drug Overdose Mortality **eAppendix 4.** Equation for Estimator of the Total Persons With HCV Infection in Each US State **eAppendix 5.** Description of Analytic Weight Derivation **eAppendix 6.** Further Descriptions of Analyses for Additional Populations Not in NHANES Sampling Frame **eFigure 1.** Conceptual Overview of Method for Estimating Hepatitis C Virus (HCV) RNA Prevalence in US States **eFigure 2.** Schematic for Levels of Specificity in Coding Injection-Related Overdose Deaths in the National Vital Statistics System **eTable 1.** National Distribution of Drug Deaths by Intentionality and Narcotic Involvement, National Vital Statistics System, 2013-2016 **eTable 2.** State-Level Total Drug Deaths and Narcotic Deaths by Intentionality, National Vital Statistics System 2013-2016 **eTable 3.** Values of Three Analytic Weighting Schemas **eTable 4.** Estimated Prevalence of HCV Antibody, NHANES 1999-2012 and 2013-2016, by Birth Cohort **eTable 5.** Sensitivity Analysis of Results Under Two Assumptions for Cumulative Mortality for 1945-1969 Birth Cohort, Among Population Included in NHANES Sampling Frame **eTable 6.** Summary of Additional Population Analytic Considerations **eTable 7.** Comparison Between Primary and Alternative Approach to Additional Population Estimates **eReferences**
