# Supplementary material for: Prevalence of Hepatitis C Virus Infection in US States and the District of Columbia, 2013 to 2016
Source: JAMA Netw Open. 2018 Dec 21;1(8):e186371. doi: 10.1001/jamanetworkopen.2018.6371 (PMC6324373; doi:10.1001/jamanetworkopen.2018.6371)
Supplement: Supplement. — eAppendix 1. NHANES Methodological Details eAppendix 2. Imputation for Missing Poverty Data in the National Health and Nutrition Examination Survey (NHANES) and the American Community Survey (ACS) eAppendix 3. Drug Overdose Mortality eAppendix 4. Equation for Estimator of the Total Persons With HCV Infection in Each US State eAppendix 5. Description of Analytic Weight Derivation eAppendix 6. Further Descriptions of Analyses for Additional Populations Not in NHANES Sampling Frame eFigure 1. Conceptual Overview of Method for Estimating Hepatitis C Virus (HCV) RNA Prevalence in US States eFigure 2. Schematic for Levels of Specificity in Coding Injection-Related Overdose Deaths in the National Vital Statistics System eTable 1. National Distribution of Drug Deaths by Intentionality and Narcotic Involvement, National Vital Statistics System, 2013-2016 eTable 2. State-Level Total Drug Deaths and Narcotic Deaths by Intentionality, National Vital Statistics System 2013-2016 eTable 3. Values of Three Analytic Weighting Schemas eTable 4. Estimated Prevalence of HCV Antibody, NHANES 1999-2012 and 2013-2016, by Birth Cohort eTable 5. Sensitivity Analysis of Results Under Two Assumptions for Cumulative Mortality for 1945-1969 Birth Cohort, Among Population Included in NHANES Sampling Frame eTable 6. Summary of Additional Population Analytic Considerations eTable 7. Comparison Between Primary and Alternative Approach to Additional Population Estimates eReferences [file jamanetwopen-1-e186371-s001.pdf]

Supplementary Online Content

Rosenberg ES, Rosenthal EM, Hall EW, et al. Prevalence of hepatitis C virus infection in US states and the District of Columbia, 2013 to 2016. *JAMA Netw Open*. 2018;1(8):e186371. doi:10.1001/jamanetworkopen.2018.6371

eAppendix 1. NHANES Methodological Details

eAppendix 2. Imputation for Missing Poverty Data in the National Health and Nutrition Examination Survey (NHANES) and the American Community Survey (ACS)

eAppendix 3. Drug Overdose Mortality

eAppendix 4. Equation for Estimator of the Total Persons With HCV Infection in Each US State

eAppendix 5. Description of Analytic Weight Derivation

eAppendix 6. Further Descriptions of Analyses for Additional Populations Not in NHANES Sampling Frame

eFigure 1. Conceptual Overview of Method for Estimating Hepatitis C Virus (HCV) RNA Prevalence in US States

eFigure 2. Schematic for Levels of Specificity in Coding Injection-Related Overdose Deaths in the National Vital Statistics System

eTable 1. National Distribution of Drug Deaths by Intentionality and Narcotic Involvement, National Vital Statistics System, 2013-2016

eTable 2. State-Level Total Drug Deaths and Narcotic Deaths by Intentionality, National Vital Statistics System 2013-2016

eTable 3. Values of Three Analytic Weighting Schemas

eTable 4. Estimated Prevalence of HCV Antibody, NHANES 1999-2012 and 2013-2016, by Birth Cohort

eTable 5. Sensitivity Analysis of Results Under Two Assumptions for Cumulative Mortality for 1945-1969 Birth Cohort, Among Population Included in NHANES Sampling Frame

eTable 6. Summary of Additional Population Analytic Considerations

eTable 7. Comparison Between Primary and Alternative Approach to Additional Population Estimates

eReferences

© 2018 Rosenberg ES et al. *JAMA Network Open.*

This supplementary material has been provided by the authors to give readers additional information about their work.

© 2018 Rosenberg ES et al. *JAMA Network Open.*

eAppendix 1: NHANES methodological details

This analysis utilized NHANES data from 1999-2016. Response rates for NHANES were: 76% in 1999-2000, 80% in 2001-2002, 76% in 2003-2004, 77.36% in 2005-2006, 75.4% in 2007-2008, 77.3% in 2009-2010, 69.5% in 2011-2012, 68.5% in 2013-2014, and 58.7% in 2015-2016.[1] Written consent was obtained for participants aged 12 and older and parents or guardians of participants younger than 18, and written assent was obtained for youth 7 to 11 years old. NHANES protocol was approved by the National Center for Health Statistics Research Ethics Review Board.[2]

eAppendix 2: Imputation for missing poverty data in the National Health and Nutrition Examination Survey (NHANES) and the American Community Survey (ACS)

NHANES collects data on household and family income as part of the demographic questionnaire administered to all respondents. NHANES uses annual poverty guidelines that vary by state and family size from the Department of Health and Human Services to calculate a ratio of family income to the poverty level. Of all NHANES respondents from 1999-2016 (N=75,974), 8.30% (n=6,303) were missing data for poverty ratio.

We used a multiple imputation regression process to impute income-to-poverty ratio for all observations with missing values. First, we categorized all values of income-to-poverty ratio into: below the poverty level, 1.0-1.9 times the poverty level and ≥2 times the poverty level. Second, we used polytomous logistic regression to model the predicted probability of being within each income-to-poverty ratio categorization using the same race, sex and birth year categories from the primary analysis as predictor variables. This resulted in income-to-poverty ratio probability distributions specific to the covariate pattern of each individual observation. For observations that were missing income-to-poverty ratio values, we randomly drew a poverty-to-income value (from the individual defined distribution) to be imputed. Each run of the Monte Carlo simulation incorporated a new random draw from each individual income-to-poverty ratio distribution. The final analytic dataset used in the primary analysis included 47,387 respondents 18 years of age or older with non-missing HCV RNA test results. In the final analytic dataset, 3,931 (8.30%) or respondents had imputed income-to-poverty ratio values.

Data used to generate ACS population estimates also contain comparable income-to-poverty level ratios for individual respondents. Of the 8,369,036 adult respondents who are not on active military duty in the 2012-2016 ACS PUMS dataset (the denominators for national NHANES analyses), 1.98% (n=237,600) were missing values for income-to-poverty level ratio. To impute these values, we followed an analogous process as the NHANES imputation model. We fit a polytomous regression model to predict the income-to-poverty level ratio distribution using state, race, sex and birth year as predictor variables. Each run of the Monte Carlo simulation randomly selected an income-to-poverty level value from the individual distributions for observations with missing values.

eAppendix 3: Drug overdose mortality

Injection drug use is the most commonly reported risk factor for acute HCV.[3] Death certificate records submitted to the National Vital Statistics System (NVSS) contain useful data on drug overdose deaths that may specifically inform HCV-related risks. However, injection-specific drug use death, which is most ideal to signal HCV infection due to injection drug use, is neither reported as an *ICD-10* code on death certificates or consistently in the open-text portions of certificates.[4] We depict in eFigure 2 a conceptual model of the levels of detail available in mortality data in order to reach the underlying ideal construct of injection-related deaths. Not all of these levels are readily available from the NVSS Multiple Cause of Death microdata files and we present our analytic case-definition as the optimal combination of specificity and sensitivity, given this challenge.

Level 1: Overdose deaths by state

Level 1 depicts the most basic information regarding deaths with underlying cause of death drug poisoning codes available from NVSS mortality data. Drug poisoning *ICD-10* codes are classified into four categories of intentionality [unintentional (X40-44), suicide (X60-64), homicide (X65), and undetermined intent (Y10-Y14)]. Many publications that describe the opioid epidemic focus on all drug poisonings of all intentionalities.[5-7] The case definition described in the Methods focuses on overdoses of unintentional and undetermined intent, which have been the focus of additional recent assessments of opioid mortality.[8,9] Overdoses of undetermined intent are included to increase the sensitivity of this measure as it would include potentially accidental or non-accidental overdoses for

which there was not enough information to record these otherwise. There were some differences in the proportion of overdoses of undetermined intent by state, which are shown in eTable 1.

We explored the potential for some drug deaths coded as suicides to be accidental overdoses. The proportion of narcotic and unknown drug deaths coded as suicides varies by state (eTable 2). Since these vary only modestly, we did not include suicides in the primary analysis. Additionally, drug intoxication does not result in a majority of suicide deaths, relative to other (more violent) methods.[10] It is actually possible that our inclusion of deaths of undetermined intent includes misclassified suicides that did not have enough evidence to be reported as suicides.[10,11]

Level 2: Overdose deaths by drug class by state

Level 2 depicts a bit more detail that is available in NVSS mortality data with regards to drug overdose deaths. Within each category of intentionality, *ICD-10* codes are separated by drug class. These classes include: poisoning by and exposure to non-opioid analgesics, antipyretics, and antirheumatics; poisoning by and exposure to antiepileptic, sedative-hypnotic, antiparkinsonism and psychotropic drugs, not elsewhere classified; poisoning by and exposure to narcotics and psychodysleptics (hallucinogens), not elsewhere classified; poisoning by and exposure to other drugs acting on the autonomic nervous system; and poisoning by and exposure to other and unspecified drugs, medicaments, and biological substances. For this analysis, our definition was restricted to deaths due to poisoning by narcotics and psychodysleptics (X42, Y12) and exposure to other and unspecified drugs (X44, Y14).

Drug overdoses due to narcotics were included because this drug class includes cannabis, cocaine, codeine, heroin, methadone, morphine, and opium. While not all of these drugs are typically administered via injection, this class provides a more specific definition than merely using all drugs.

Death investigation and drug toxicology processes vary by state.[12-14] In order to account for this variation, and to provide a more sensitive definition, we included overdoses due to other and unspecified drugs.[8,15]

Level 3: Overdose deaths by specific drugs by state

The third level describes specific drugs that are more likely to be used via injection than other drugs. NVSS mortality data includes specific drug toxicology codes (T codes) for heroin, natural and semisynthetic opioids (morphine, codeine, hydrocodone, and oxycodone), methadone, synthetic opioids excluding methadone (fentanyl, fentanyl analogs, and tramadol), cocaine, and psychostimulants with abuse potential (methamphetamine, amphetamine, Ritalin, caffeine, and ecstasy). While inclusion of T codes for injection-related drugs such as heroin and synthetic opioids excluding methadone would be an improved signal of injection-related overdose, the toxicology completion regarding specific drug codes on death certificates varies greatly by state and by year.[12-14,16,17]

A second issue with using specific drug codes for this analysis is that these are not mutually exclusive. Many drugs, particularly heroin and fentanyl, are found together in toxicology and subsequent death certificates.[18] This becomes an issue since some drugs (i.e., fentanyl) have higher fatality rates from injection than others, and some drugs are more frequently used via injection than other routes of administration.[19,20] This is particularly important for assessing the geographic distribution of overdose deaths since the distribution of fentanyl varies, in part due to the relative ease of incorporating fentanyl into the white powder heroin supply east of the Mississippi River, compared to black tar heroin.[21] These have not been incorporated into the present analysis due to the variation of completion by state,[22] but this is a critical issue that should be explored in future research, in order to reduce biases introduced by non-specificity for injected drugs and by spatial heterogeneity in highly-lethal substances such as fentanyl.

Level 4: Overdose deaths by specific drugs and injection status by state

Finally, for the fourth level, the ideal measurement of injection-related overdose is overdose by drug by injection. The most relevant literature-based estimate of opioid overdose deaths attributable to injection is not generalizable to all states.[23] This is the ideal measure to use for those born after 1970 for the HCV work. However, due to the lack of literature as well as the above-mentioned limitations in the specific drugs, we cannot achieve this level of detail.

© 2018 Rosenberg ES et al. *JAMA Network Open.*

eAppendix 4: Equation for Estimator of the Total Persons With HCV Infection in Each US State

$$\hat{T}_i = \sum_{j=1}^{J=12}\left[(N_{ij} \times \hat{\mu}_j) \times \left([w_j]\frac{\hat{\theta}_{ij}}{\left(\frac{\sum_{i=1}^{I}(\hat{\theta}_{ij} \times N_{ij}^*)}{\sum_{i=1}^{I} N_{ij}^*}\right)} + [1 - w_j]\frac{\hat{\varphi}_{ij}}{\left(\frac{\sum_{i=1}^{I}(\hat{\varphi}_{ij} \times N_{ij}^*)}{\sum_{i=1}^{I} N_{ij}^*}\right)}\right)\right]$$

Where:
i = states 1 to I (I=51)
j = stratum 1 to J (J = 12), formed by combination of sex (2), birth cohort (3), race (2)
$\hat{T}_i$ = Estimated total persons with HCV RNA, in state i
$\hat{\mu}_j$ = Estimated weighted HCV prevalence, in stratum j
$\hat{\theta}_{ij}$ = Estimated probability of HCV-related mortality, in stratum j of state i
$\hat{\varphi}_{ij}$ = Estimated probability of narcotic overdose mortality, in stratum j of state i
w_j = Mortality ratio weight for stratum j
N_{ij}= Adult population in stratum j of state i
N_{ij}^*= Adult person-years in stratum j of state i

The above equation details our estimator for the total persons with HCV in each state i ($\hat{T}_i$) in the NHANES population. Within 12 strata J representing above-defined levels of sex, race/ethnicity, and birth year, we computed the standardization estimate $(N_{ij} \times \hat{\mu}_j)$, where N_{ij} is the 2016 ACS population in the state's stratum ("state-stratum") and $\hat{\mu}_j$ the national 2013-2016 HCV RNA prevalence in stratum j by direct estimation from a weighted logistic regression model of NHANES, which included terms for these strata, era (1999-2012, 2013-2016), and poverty. To yield standardized estimates for the 12 demographic strata that accounted for poverty, we weighted logistic model estimates according to the ACS poverty distribution for the 12 strata in each state.

Next, we estimated the state-stratum-specific likelihood of HCV-related mortality ($\hat{\theta}_{ij}$), using a logistic model of NVSS-derived mortality counts, per person-years (N_{ij}^*), that approximated full-stratification with main effects for state, sex, race/ethnicity, birth cohort, era; two-way interactions for state by each sex/ethnicity, race, birth cohort, and era; two-way and three-way interactions for each combination of sex, race/ethnicity, birth cohort, and era; and four-way interaction of sex, race/ethnicity, birth cohort, and era. These $\hat{\theta}_{ij}$ were divided by the national stratum-specific average, yielding a mortality ratio for the state-stratum. This process was repeated for the narcotic overdose mortality ($\hat{\varphi}_{ij}$). The two mortality ratios per stratum were averaged according to weights w_j (values described in the main manuscript text and eAppendix 5) and then multiplied by the standardization-based value to yield adjusted totals $\hat{T}_{ij}$. Summing these across all 12 state-strata yielded $\hat{T}_i$, which when divided by the ACS state population N_i yielded the estimated prevalence rate.

eAppendix 5: Description of analytic weight derivation

To represent the spatial distribution of both older prevalent HCV infections (those existing during 1999-2012) and newer HCV infections (during 2013-2016) resulting from injection drug use, we separately modeled mortality rates from HCV infection and narcotic overdose. Mortality rates were used to calculate state-level mortality ratios for both HCV infection and narcotic overdose. Within each age group (defined by birth year), we calculated a single weighted state-level mortality ratio.

For the first weighting scenario, we only used state-level mortality ratios from the HCV death model in order to compare to our previous method (eTable 3, results depicted in Figure 2).

© 2018 Rosenberg ES et al. *JAMA Network Open.*

For the second scenario, we used available data and expert knowledge of HCV epidemiology to derive the weights. First, we assumed all HCV infections among persons born <1945 are a result of older exposure and used a weight of 1.0 for that age group (w_1). For the other two age groups, we used trends of HCV antibody prevalence in NHANES data to make inferences and assumptions about HCV incidence from 2013-2016. For persons born ≥1970, we estimated there were 411,449 persons with a history of infection (HCV antibody) prior to 2013 and 1,253,938 after 2013 (eTable 4). We assumed ~0% mortality for this age group during this time frame, suggesting 37.8% of persons with HCV exposure in this age group acquired HCV prior to 2013 and 62.2% thereafter. This is similar to other observations around 70%.[24] Therefore, we used a weight of 0.378 for the HCV death state effect ratio for persons born ≥1970 (w_3).

The estimated number of persons born from 1945-1969 with HCV antibody did not meaningfully change between 1999-2012 (n=3,092,027) and 2013-2016 (n=2,848,019), which suggests that total number of incident infections is approximately equal to the total number of persons in this cohort who died between the midpoint of 1999-2012 to the midpoint of 2013-2016. Thus, estimating the cumulative death rate of persons born 1945-1969 with HCV antibody would provide an estimate of incidence to inform our weighting. Using a life-table approach incorporating all-cause mortality NVSS data and ACS population sizes for those born 1945-1969, we estimated the all-cause likelihood of death in the general population to be 0.047 between 1/1/2007 and 12/31/2014. We assumed persons in this age group who spontaneously clear their HCV infection or do not have a positive HCV RNA diagnosis experience the same death rate as the general population. From 2013-2016 NHANES data, 53.6% of persons born between 1945-1969 with HCV antibodies had a positive HCV RNA test and 62.0% of HCV RNA+ individuals had an HCV diagnosis. A previous paper reported that persons born between 1945-1964 with an HCV RNA diagnosis had an estimated mortality of 0.282.[25] From this, we used the following calculation to estimate the mortality rate for persons born 1945-1969 with HCV antibody:

$$[0.282 \times (0.536 \times 0.620)] + \left[0.047 \times \left(1 - (0.536 \times 0.620)\right)\right] = 0.125$$

Based on the assumption of a stable number of HCV infections within this age group, we used a weight of 0.875 for persons born 1945-1969 (w_3).

Persons with HCV antibody, but who are not currently infected, and those who are currently infected but are not diagnosed (and presumably relatively asymptomatic), may experience death rates higher than the background mortality rates, although such data are unavailable. Recognizing that this age group (persons born 1945-1969) has the highest HCV burden and may disproportionally impact prevalence results, we conducted a sensitivity analysis examining a third scenario that considered an overall mortality rate of 20% for person with HCV antibody (w_3 = 0.80) (eTable 5).

eAppendix 6: Further descriptions of analyses for additional populations not in NHANES sampling frame
This analysis used the results of a literature review from Hofmeister et al.,[26] which used articles that reported HCV prevalence from 1/1/2013-12/31/2017. Search terms used for the incarcerated population were ("hepatitis C" or "HCV") and ("prison" or "jail" or "correctional") and for the homeless population were ("hepatitis C" or "HCV") and ("homeless" or "homeless persons" or "housing unstable" or "housing insecure"). Details on motivation for additional populations and prevalence and population size sources used in Hofmeister et al.[26] are described in eTable 6.

Alternative Approach to Additional Population Estimates

The alternative approach to estimating state-level HCV RNA prevalence among additional populations involved two steps. First, we generated a national prevalence ratio for each population component (incarcerated, unsheltered homeless, and nursing home residents) by taking the national HCV prevalence in the population component divided by the national HCV prevalence in NHANES. Then, we multiplied this national prevalence ratio by the each state's HCV prevalence in the NHANES population and each state's population size of each population component. This provided an estimate of HCV infections among additional populations that reflects each state's underlying HCV

© 2018 Rosenberg ES et al. *JAMA Network Open.*

prevalence rather than the national HCV estimate. This assumes that the state epidemics are echoed in these additional populations.

Full results including both the primary approach for the additional population estimate and the alternative are shown in eTable 7. There was a median difference in prevalence between methods 1 and 2 of 0.004% (relative multiplicative change of -0.5%).

© 2018 Rosenberg ES et al. *JAMA Network Open.*

eFigure 1: Conceptual overview of method for estimating Hepatitis C virus (HCV) RNA prevalence in US states

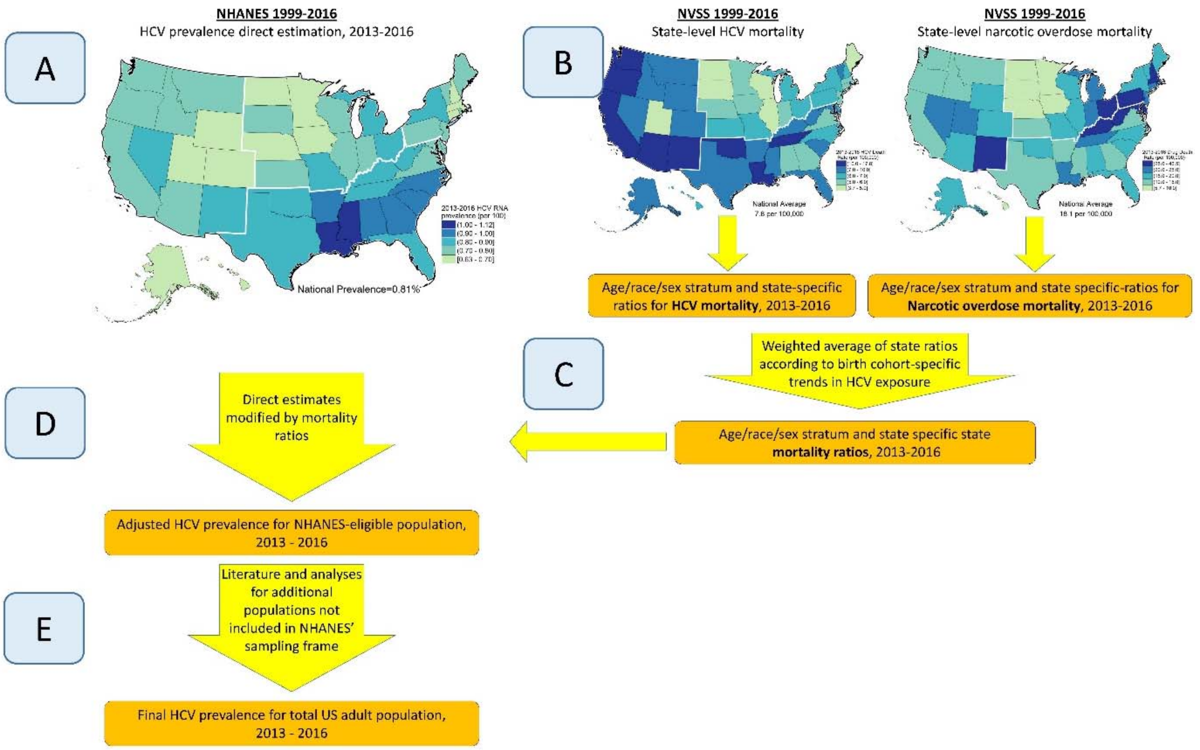

We used a multistep, statistical approach that first generated estimates for each state using National Health and Nutrition Examination Survey (NHANES) national prevalence in sex, race, birth cohort, and poverty strata (1A). To represent the spatial distribution of older and recent infections respectively, we separately modeled mortality rates from HCV infection and narcotic overdose in the National Vital Statistics System (NVSS), yielding stratified state-level mortality ratios (1B). We weighted these ratios according to birth cohort-specific trends in HCV exposure history (1C) and used them to adjust initial NHANES-based estimates (1D). Finally, we estimated additional infections among populations not included in NHANES' sampling frame, by applying literature-based estimates of prevalence in these groups to state-specific population estimates (1E).

eFigure 2: Schematic for levels of specificity in coding injection-related overdose deaths in the National Vital Statistics System

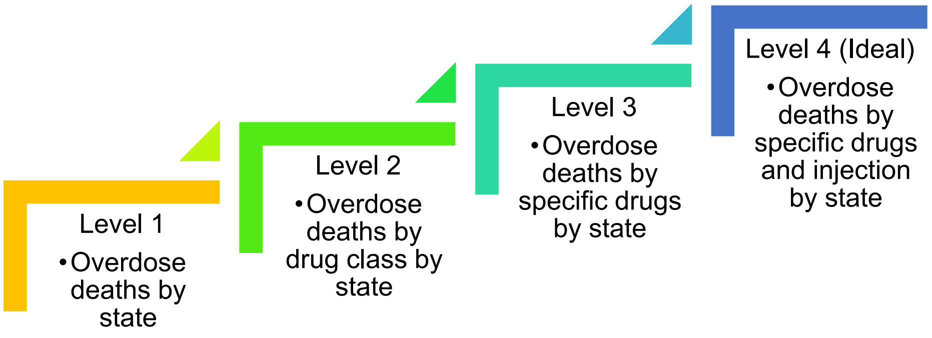

© 2018 Rosenberg ES et al. *JAMA Network Open.*

eTable 1. National distribution of drug deaths by intentionality and narcotic involvement, National Vital Statistics System, 2013-2016

Intentionality of death and drug class[a]	n (%)
All-intention deaths, all drug classes[b]	221,710 (100%)
• Homicide	497 (0.22%)
• Unintentional, suicide, undetermined deaths	221,213 (99.8%)
o Narcotics and unspecified drugs	195,134 (88%)
▪ Suicide deaths	17,017 (9%)
➢ *Narcotics only*	*2,869 (17%)*
➢ *Unspecified drugs only*	*14162 (83%)*
▪ Unintentional and undetermined cause deaths [c]	178,122 (91%)
➢ Unintentional deaths	166,822 (85%)
• *Narcotics only*	*82,288 (49%)*
• *Unspecified drugs only*	*84,609 (51%)*
➢ Undetermined deaths	11,300 (6%)
• *Narcotics only*	*5,449 (48%)*
• *Unspecified drugs only*	*5,857 (52%)*

[a] Drug intentions are defined by ICD-10 codes:

- Unintentional: X40-X44
- Intentional self-poisoning (suicide): X60-X64
- Homicide includes: X85
- Undetermined intent: Y10-Y14

Drug classes are defined by ICD-10 codes:

- Poisoning by and exposure to nonopioid analgesics, antipyretics, and antirheumatics: X40, X60, Y10
- Poisoning by and exposure to antiepileptic, sedative-hypnotic, antiparkinsonism and psychotropic drugs, not elsewhere classified: X41, X61, Y11
- Poisoning by and exposure to narcotics and psychodysleptics (hallucinogens), not elsewhere classified: X42, X62, Y12
- Poisoning by and exposure to other drugs acting on the autonomic nervous system: X43, X63, Y13
- Poisoning by and exposure to other and unspecified drugs, medicaments, and biological substances: X44, X64, Y14

[b] This is how many publications often describe drug overdoses[5-7]

[c] This is the definition used in this analysis.

© 2018 Rosenberg ES et al. *JAMA Network Open.*

eTable 2. State-level total drug deaths and narcotic deaths by intentionality, National Vital Statistics System 2013-2016

	Deaths from all drugs (unintentional, suicide, and undetermined intent)	Deaths from narcotic or unspecified drugs			
		Unintentional, suicide, and undetermined intent	Unintentional only[a]	Suicide only[a]	Undetermined intent only[a]
**Total**	221,213	195,134	166,822 (85%)	17,017 (9%)	11,300 (6%)
**State**					
Alabama	2,958	2,767	2,474 (89%)	169 (6%)	124 (4%)
Alaska	527	450	378 (84%)	49 (11%)	23 (5%)
Arizona	5,531	4,390	3,470 (79%)	536 (12%)	384 (9%)
Arkansas	1,610	1,264	926 (73%)	178 (14%)	160 (13%)
California	21,077	15,322	12,975 (85%)	2,000 (13%)	348 (2%)
Colorado	3,823	3,229	2,564 (79%)	541 (17%)	125 (4%)
Connecticut	3,027	2,865	2,627 (92%)	200 (7%)	38 (1%)
Delaware	860	811	716 (88%)	53 (7%)	42 (5%)
District of Columbia	613	574	497 (87%)	24 (4%)	53 (9%)
Florida	13,777	12,641	11,123 (88%)	1,387 (11%)	131 (1%)
Georgia	5,227	4,517	4,047 (90%)	374 (8%)	97 (2%)
Hawaii	754	422	308 (73%)	69 (16%)	45 (11%)
Idaho	945	748	534 (71%)	133 (18%)	81 (11%)
Illinois	8,059	7,448	6,604 (89%)	571 (8%)	273 (4%)
Indiana	5,428	5,052	4,241 (84%)	384 (8%)	427 (8%)
Iowa	1,341	1,043	813 (78%)	173 (17%)	57 (5%)
Kansas	1,506	1,202	947 (79%)	187 (16%)	68 (6%)
Kentucky	5,073	4,769	4,325 (91%)	198 (4%)	246 (5%)
Louisiana	3,789	3,533	3,114 (88%)	182 (5%)	237 (7%)
Maine	1,052	963	854 (89%)	91 (9%)	18 (2%)
Maryland	5,399	5,107	1,396 (27%)	214 (4%)	3,497 (68%)
Massachusetts	6,493	6,208	5,857 (94%)	267 (4%)	84 (1%)
Michigan	7,915	7,522	6,001 (80%)	580 (8%)	941 (13%)
Minnesota	2,426	1,985	1,658 (84%)	248 (12%)	79 (4%)
Mississippi	1,467	1,316	1,147 (87%)	108 (8%)	61 (5%)
Missouri	4,791	4,119	3,540 (86%)	385 (9%)	194 (5%)
Montana	553	444	283 (64%)	83 (19%)	78 (18%)
Nebraska	552	438	349 (80%)	70 (16%)	19 (4%)
Nevada	2,865	2,152	1,794 (83%)	311 (14%)	47 (2%)
New Hampshire	1,487	1,403	1,273 (91%)	101 (7%)	29 (2%)
New Jersey	6,334	6,066	5,628 (93%)	333 (5%)	105 (2%)
New Mexico	2,195	1,828	1,588 (87%)	205 (11%)	35 (2%)
New York	11,543	10,845	9,458 (87%)	779 (7%)	608 (6%)
North Carolina	6,289	5,840	5,109 (87%)	580 (10%)	151 (3%)
North Dakota	229	192	155 (81%)	22 (11%)	15 (8%)

© 2018 Rosenberg ES et al. *JAMA Network Open.*

	Deaths from all drugs (unintentional, suicide, and undetermined intent)	Deaths from narcotic or unspecified drugs			
		Unintentional, suicide, and undetermined intent	Unintentional only[a]	Suicide only[a]	Undetermined intent only[a]
Ohio	13,397	12,777	11,975 (94%)	602 (5%)	200 (2%)
Oklahoma	3,360	2,401	2,098 (87%)	172 (7%)	131 (5%)
Oregon	2,129	1,650	1,239 (75%)	273 (17%)	138 (8%)
Pennsylvania	13,336	12,852	11,730 (91%)	786 (6%)	337 (3%)
Rhode Island	1,167	1,090	1,002 (92%)	70 (6%)	18 (2%)
South Carolina	3,203	2,874	2,600 (90%)	226 (8%)	48 (2%)
South Dakota	268	192	135 (70%)	46 (24%)	11 (6%)
Tennessee	5,860	5,319	4,742 (89%)	364 (7%)	213 (4%)
Texas	11,732	9,615	8,314 (86%)	979 (10%)	322 (3%)
Utah	2,622	2,276	1,567 (69%)	305 (13%)	405 (18%)
Vermont	433	389	311 (80%)	42 (11%)	36 (9%)
Virginia	4,401	3,963	3,471 (88%)	368 (9%)	124 (3%)
Washington	4,496	3,541	2,955 (83%)	460 (13%)	126 (4%)
West Virginia	3,095	2,847	2,601 (91%)	126 (4%)	120 (4%)
Wisconsin	3,769	3,503	3,006 (86%)	363 (10%)	134 (4%)
Wyoming	430	370	303 (82%)	50 (14%)	17 (5%)
**Mean**	4,338	3,826	3,271	334	222
**Median**	3,095	2,847	2,474	214	120
**Lower Quartile**	1,254	1,067	890	105	46
**Upper Quartile**	5,480	5,080	4,283	385	207

[a] Denominator for percentage is deaths from narcotics and unspecified drugs of unintentional, suicide, and undetermined intent.

© 2018 Rosenberg ES et al. *JAMA Network Open.*

eTable 3: Values of three analytic weighting schemas

Scenario<br>Birth Year	Weight w_j applied to HCV death state effect (standardized ratio)	Weight $(1 - w_j)$ applied to drug overdose death state effect (standardized ratio)
1. ICD-10 Codes for HCV Only		
<1945	100%	0%
1945-1969	100%	0%
≥1970	100%	0%
2. ICD-10 Codes for HCV and Narcotic Overdose *(primary analysis)*		
<1945	100%	0%
1945-1969	87.5%	12.5%
≥1970	37.8%	62.2%
3. ICD-10 Codes for HCV and Narcotic Overdose *(sensitivity analysis)*		
<1945	100%	0%
1945-1969	80.0%	20.0%
≥1970	37.8%	62.2%

Abbreviations: HCV, hepatitis C virus; ICD-10, *International Classification of Diseases, Tenth Revision*

eTable 4: Estimated prevalence of HCV antibody, NHANES 1999-2012 and 2013-2016, by birth cohort

		2005-2007 ACS		NHANES anti-HCV+						
**1999-2012**		**N**		**Per 100**	**95% CI**			**n**	**95%CI**	
<1945		43,453,450		0.66	0.50	0.88		287,749	216,876	381,521
1945-1969		100,776,581		3.07	2.70	3.48		3,092,027	2,721,572	3,511,056
≥1970		76,820,211		0.54	0.38	0.75		411,449	292,378	578,533
		**2012-2016 ACS**		**NHANES anti-HCV+**						
**2013-2016**		**N**		**Per 100**	**95% CI**			**n**	**95%CI**	
<1945		29,693,961		0.52	0.28	0.94		152,983	83,648	279,272
1945-1969		97,702,202		2.92	2.38	3.57		2,848,019	2,321,697	3,489,239
≥1970		113,756,485		0.96	0.69	1.32		1,087,171	783,782	1,506,363

Abbreviations: HCV, hepatitis C virus; NHANES, National Health and Nutrition Examination Survey; ACS, American Community Survey; anti-HCV, hepatitis C virus antibody; CI, confidence interval

eTable 5. Sensitivity analysis of results under two assumptions for cumulative mortality for 1945-1969 birth cohort, among population included in NHANES sampling frame

		Primary Analysis Results (12.5% mortality)						Sensitivity Analysis Results (20.0% mortality)					
**State**	**ACS 2012-2016[a]**	**HCV RNA+[b]**	**95% CI**		**%**	**(95% CI)**		**HCV RNA+[b]**	**95% CI**		**% [c]**	**(95% CI)**	
Alabama	3,671,100	26,100	(23,100 -	29,600)	0.71	(0.63 -	0.81)	26,100	(23,100 -	29,600)	0.71	(0.63 -	0.81)
Alaska	542,500	4,700	(3,900 -	5,700)	0.86	(0.72 -	1.05)	4,600	(3,800 -	5,600)	0.85	(0.70 -	1.03)
Arizona	5,020,500	55,300	(48,000 -	64,100)	1.10	(0.96 -	1.28)	54,800	(47,600 -	63,500)	1.09	(0.95 -	1.26)
Arkansas	2,215,500	19,100	(16,800 -	21,800)	0.86	(0.76 -	0.99)	18,600	(16,400 -	21,300)	0.84	(0.74 -	0.96)
California	29,160,200	288,500	(253,500 -	331,800)	0.99	(0.87 -	1.14)	281,200	(247,200 -	323,100)	0.96	(0.85 -	1.11)
Colorado	4,057,000	32,500	(28,000 -	38,400)	0.80	(0.69 -	0.95)	32,100	(27,600 -	37,800)	0.79	(0.68 -	0.93)
Connecticut	2,771,800	16,500	(14,200 -	19,700)	0.60	(0.51 -	0.71)	17,200	(14,700 -	20,500)	0.62	(0.53 -	0.74)
Delaware	719,400	5,600	(4,800 -	6,500)	0.78	(0.67 -	0.90)	5,700	(4,900 -	6,600)	0.79	(0.68 -	0.92)
District of Columbia	537,500	12,400	(10,500 -	14,800)	2.32	(1.95 -	2.76)	12,600	(10,600 -	15,000)	2.35	(1.98 -	2.80)
Florida	15,620,600	133,200	(117,700 -	152,100)	0.85	(0.75 -	0.97)	132,700	(117,300 -	151,600)	0.85	(0.75 -	0.97)
Georgia	7,465,900	46,400	(41,300 -	52,300)	0.62	(0.55 -	0.70)	46,500	(41,400 -	52,400)	0.62	(0.56 -	0.70)
Hawaii	1,094,200	5,700	(4,700 -	7,000)	0.52	(0.43 -	0.64)	5,600	(4,600 -	6,800)	0.51	(0.42 -	0.62)
Idaho	1,187,300	9,900	(8,400 -	11,800)	0.84	(0.71 -	0.99)	9,600	(8,200 -	11,400)	0.81	(0.69 -	0.96)
Illinois	9,703,700	47,700	(42,200 -	54,300)	0.49	(0.44 -	0.56)	50,500	(44,700 -	57,400)	0.52	(0.46 -	0.59)
Indiana	4,915,800	35,400	(30,900 -	40,700)	0.72	(0.63 -	0.83)	36,000	(31,500 -	41,500)	0.73	(0.64 -	0.84)
Iowa	2,339,900	11,100	(9,500 -	13,100)	0.47	(0.40 -	0.56)	10,900	(9,300 -	12,900)	0.47	(0.40 -	0.55)
Kansas	2,137,000	12,600	(10,900 -	14,800)	0.59	(0.51 -	0.69)	12,500	(10,700 -	14,700)	0.58	(0.50 -	0.69)
Kentucky	3,331,500	38,600	(33,600 -	44,800)	1.16	(1.01 -	1.34)	39,600	(34,500 -	45,900)	1.19	(1.04 -	1.38)
Louisiana	3,445,000	44,900	(40,000 -	50,400)	1.30	(1.16 -	1.46)	44,400	(39,700 -	49,900)	1.29	(1.15 -	1.45)
Maine	1,058,600	6,500	(5,400 -	7,800)	0.61	(0.51 -	0.74)	6,600	(5,600 -	8,000)	0.63	(0.53 -	0.75)
Maryland	4,547,800	37,300	(32,700 -	43,100)	0.82	(0.72 -	0.95)	38,600	(33,800 -	44,600)	0.85	(0.74 -	0.98)
Massachusetts	5,283,400	35,800	(30,600 -	42,500)	0.68	(0.58 -	0.80)	37,200	(31,900 -	44,200)	0.70	(0.60 -	0.84)
Michigan	7,578,400	62,800	(55,800 -	70,900)	0.83	(0.74 -	0.94)	64,400	(57,200 -	72,700)	0.85	(0.76 -	0.96)
Minnesota	4,115,000	22,300	(19,400 -	26,000)	0.54	(0.47 -	0.63)	22,000	(19,200 -	25,600)	0.53	(0.47 -	0.62)
Mississippi	2,205,500	19,600	(17,500 -	22,200)	0.89	(0.79 -	1.01)	19,300	(17,100 -	21,800)	0.87	(0.78 -	0.99)
Missouri	4,575,700	35,200	(31,100 -	40,200)	0.77	(0.68 -	0.88)	35,700	(31,400 -	40,700)	0.78	(0.69 -	0.89)
Montana	787,100	6,800	(5,700 -	8,000)	0.86	(0.73 -	1.02)	6,600	(5,600 -	7,800)	0.84	(0.71 -	0.99)
Nebraska	1,391,400	6,900	(6,000 -	8,200)	0.50	(0.43 -	0.59)	6,700	(5,800 -	7,900)	0.48	(0.41 -	0.57)
Nevada	2,148,500	19,300	(16,800 -	22,400)	0.90	(0.78 -	1.04)	19,500	(17,000 -	22,700)	0.91	(0.79 -	1.06)
New Hampshire	1,046,300	7,200	(5,900 -	8,900)	0.69	(0.57 -	0.85)	7,400	(6,100 -	9,200)	0.71	(0.58 -	0.88)
New Jersey	6,810,300	43,400	(37,900 -	50,300)	0.64	(0.56 -	0.74)	44,200	(38,600 -	51,400)	0.65	(0.57 -	0.75)
New Mexico	1,557,100	25,000	(21,600 -	29,100)	1.61	(1.39 -	1.87)	24,900	(21,500 -	29,000)	1.60	(1.38 -	1.86)

© 2018 Rosenberg ES et al. *JAMA Network Open.*

		Primary Analysis Results (12.5% mortality)						Sensitivity Analysis Results (20.0% mortality)					
**State**	**ACS 2012-2016[a]**	**HCV RNA+[b]**	**95% CI**		**% [c]**	**(95% CI)**		**HCV RNA+[b]**	**95% CI**		**% [c]**	**(95% CI)**	
New York	15,260,100	107,100	(94,900 -	121,600)	0.70	(0.62 -	0.80)	108,300	(95,900 -	123,100)	0.71	(0.63 -	0.81)
North Carolina	7,545,400	60,200	(53,600 -	68,100)	0.80	(0.71 -	0.90)	59,900	(53,300 -	67,800)	0.79	(0.71 -	0.90)
North Dakota	559,100	2,200	(1,800 -	2,800)	0.39	(0.32 -	0.50)	2,200	(1,700 -	2,700)	0.39	(0.31 -	0.49)
Ohio	8,787,100	81,500	(71,800 -	93,200)	0.93	(0.82 -	1.06)	85,200	(75,200 -	97,400)	0.97	(0.86 -	1.11)
Oklahoma	2,862,800	48,900	(42,700 -	56,500)	1.71	(1.49 -	1.97)	47,400	(41,400 -	54,700)	1.66	(1.45 -	1.91)
Oregon	3,086,200	45,700	(39,400 -	53,700)	1.48	(1.28 -	1.74)	43,500	(37,500 -	51,100)	1.41	(1.21 -	1.65)
Pennsylvania	9,888,700	84,500	(74,300 -	97,000)	0.86	(0.75 -	0.98)	87,300	(76,800 -	100,200)	0.88	(0.78 -	1.01)
Rhode Island	829,900	9,600	(8,300 -	11,400)	1.16	(1.00 -	1.37)	9,800	(8,400 -	11,600)	1.18	(1.01 -	1.40)
South Carolina	3,689,100	31,900	(28,400 -	36,100)	0.87	(0.77 -	0.98)	31,900	(28,400 -	36,000)	0.86	(0.77 -	0.98)
South Dakota	628,400	3,000	(2,500 -	3,700)	0.48	(0.39 -	0.59)	2,900	(2,400 -	3,600)	0.46	(0.38 -	0.57)
Tennessee	4,972,200	63,500	(56,200 -	72,100)	1.28	(1.13 -	1.45)	63,400	(56,100 -	72,000)	1.27	(1.13 -	1.45)
Texas	19,455,200	178,000	(157,500 -	203,100)	0.91	(0.81 -	1.04)	172,500	(152,700 -	196,600)	0.89	(0.79 -	1.01)
Utah	2,024,600	11,000	(9,300 -	13,100)	0.54	(0.46 -	0.65)	11,400	(9,700 -	13,600)	0.56	(0.48 -	0.67)
Vermont	499,100	3,500	(2,900 -	4,200)	0.70	(0.58 -	0.85)	3,500	(2,900 -	4,200)	0.69	(0.57 -	0.84)
Virginia	6,348,500	33,500	(29,400 -	38,500)	0.53	(0.46 -	0.61)	33,400	(29,400 -	38,400)	0.53	(0.46 -	0.61)
Washington	5,412,700	50,000	(43,100 -	58,900)	0.92	(0.80 -	1.09)	48,700	(42,000 -	57,400)	0.90	(0.78 -	1.06)
West Virginia	1,439,300	19,500	(16,700 -	23,000)	1.35	(1.16 -	1.60)	20,400	(17,500 -	23,900)	1.41	(1.22 -	1.66)
Wisconsin	4,384,900	24,000	(21,000 -	27,700)	0.55	(0.48 -	0.63)	24,600	(21,600 -	28,300)	0.56	(0.49 -	0.65)
Wyoming	437,600	3,200	(2,600 -	3,900)	0.73	(0.60 -	0.90)	3,200	(2,600 -	3,900)	0.73	(0.60 -	0.89)
**Total[d,e]**	**241,152,600**	**2,035,100**	**(1,803,600 -**	**2,318,000)**	**0.84**	**(0.75 -**	**0.96)**	**2,033,800**	**(1,802,400 -**	**2,316,600)**	**0.84**	**(0.75 -**	**0.96)**

[a] Population sizes are estimated as of December 2016 based on American Community Survey 5-year estimates from 2012-2016 and include noninstitutionalized adults eligible for NHANES. This estimate includes 1,288,600 active-duty military personnel ineligible for NHANES, which cannot be removed at the state-level because population sizes are unavailable by home state of personnel.
[b] Number of infected persons is calculated by multiplying the prevalence percentage estimate by the adult population size, before rounding for presentation.
[c] NHANES prevalence percentage estimates are based on results from 2013-2016 NHANES. Population size includes noninstitutionalized adults eligible for NHANES from the 2012-2016 American Community Survey.
[d] Values may not sum to total due to rounding.
[e] Results are based on a regression model that incorporates data for the time period 1999-2016 and generates estimates via simulations. Accordingly, these results do not precisely sum to previous national totals for the 2013-2016 period.[26]
Abbreviations: NHANES, National Health and Nutrition Examination Survey; ACS, American Community Survey; HCV, hepatitis C virus; CI, confidence interval

© 2018 Rosenberg ES et al. *JAMA Network Open.*

eTable 6. Summary of additional population analytic considerations

	Population features evaluated for analytic decisions			Data sources used in analysis		
**Population**	**Included in NHANES sampling frame**	**Included in ACS population size estimates used for NHANES analyses**	**Evidence of Differential HCV Risk**	**HCV prevalence source**	**Mean prevalence**	**Population-size source**
Residential, noninstitutionalized, civilian population	Yes	Yes	N/A	NHANES	0.9%	ACS, 2012 – 2016[27]
Incarcerated	No	No	Yes	Literature	10.7%[a]	Bureau of Justice Statistics, 2016[28]
Unsheltered homeless	No	No	Yes	Literature	10.8%[b]	U.S. Department of Housing and Urban Development, 2016[29]
Nursing homes	No	No	No	NHANES	0.5%	National Survey of Long Term Care Providers, 2014[30,c]
People living in AI/AN areas[d, e]	Yes	Yes	Yes	N/A	N/A	N/A
Hospitalized[e]	Yes	Yes	No	N/A	N/A	N/A
Other high risk populations (e.g., persons who inject drugs, sheltered homeless)[f]	Yes	Yes	Yes	N/A	N/A	N/A

[a] Estimated mean prevalence calculated using a random effects model with prevalence inputs from Akiyama et al.,[31] Cocoros et al.,[32] de la Flor et al.,[33] Kuncio et al.,[34] Mahowald et al.,[35] Schoenbachler et al.,[36] Stockman et al.[37] For Akiyama, de la Flor, and Kuncio, RNA prevalence was calculated as (reported HCV Antibody Prevalence) x (NHANES 2013-2016 HCV RNA prevalence), where NHANES 2013-2016 HCV RNA prevalence among antibody positives= 0.575. For Cocoros, Mahowald, Schoenbachler, and Stockman, RNA prevalence was calculated as (Number HCV RNA-Positive/Number Tested HCV RNA) x (reported HCV Antibody Prevalence).

[b] Literature prevalence from Coyle et al.[38]

[c] Scaled for population growth to 2016

[d] Residents of Native American reservations and tribal lands and Alaska Native village statistical areas

[e] Excluded from analysis due to inclusion in both NHANES (prevalence numerator) and ACS (population size denominator)

[f] For persons who inject drugs, we assessed likely bias and determined that national NHANES estimates sufficiently represented HCV prevalence in this subpopulation

Abbreviations: NHANES, National Health and Nutrition Examination Survey; ACS, American Community Survey; HCV, hepatitis C virus; AI/AN, American Indian/Alaska Native

© 2018 Rosenberg ES et al. *JAMA Network Open.*

eTable 7. Comparison between primary and alternative approach to additional population estimates

				Additional Populations Estimation: Primary Method				Additional Populations Estimation: Alternative Method				Comparison between methods	
	Population			RNA+				RNA+				Difference	
State	ACS 2012-2016[a]	Additional Populations	Total[b]	Among NHANES population[c]	In Additional Populations	Total[b]	%	Among NHANES population[c]	In Additional Populations	Total[b]	%	RNA+	%
Alabama	3,671,100	65,600	3,736,700	26,100	4,600	30,700	0.82	26,100	3,900	29,900	0.80	732	0.02
Alaska	542,500	5,500	548,000	4,700	500	5,200	0.95	4,700	500	5,200	0.95	(10)	(0.00)
Arizona	5,020,500	70,000	5,090,500	55,300	6,300	61,500	1.21	55,300	8,200	63,400	1.25	(1,889)	(0.04)
Arkansas	2,215,500	43,200	2,258,700	19,100	2,700	21,800	0.97	19,100	2,800	21,900	0.97	(60)	(0.00)
California	29,160,200	384,500	29,544,700	288,500	30,400	318,900	1.08	288,500	35,500	324,000	1.10	(5,119)	(0.02)
Colorado	4,057,000	51,500	4,108,500	32,500	3,800	36,300	0.88	32,500	3,600	36,100	0.88	190	0.00
Connecticut	2,771,800	40,900	2,812,700	16,500	1,800	18,300	0.65	16,500	1,300	17,800	0.63	510	0.02
Delaware	719,400	11,100	730,500	5,600	700	6,300	0.86	5,600	700	6,300	0.86	57	0.01
District of Columbia	537,500	4,800	542,400	12,400	200	12,700	2.34	12,400	700	13,100	2.42	(414)	(0.08)
Florida	15,620,600	239,600	15,860,200	133,200	17,800	151,000	0.95	133,200	18,000	151,200	0.95	(200)	(0.00)
Georgia	7,465,900	131,700	7,597,700	46,400	10,500	56,800	0.75	46,400	7,700	54,100	0.71	2,776	0.04
Hawaii	1,094,200	13,200	1,107,400	5,700	1,000	6,700	0.60	5,700	600	6,300	0.57	391	0.04
Idaho	1,187,300	15,900	1,203,300	9,900	1,300	11,200	0.93	9,900	1,300	11,200	0.93	14	0.00
Illinois	9,703,700	138,700	9,842,400	47,700	7,100	54,900	0.56	47,700	4,100	51,800	0.53	3,015	0.03
Indiana	4,915,800	84,300	5,000,100	35,400	4,900	40,200	0.80	35,400	4,200	39,500	0.79	705	0.01
Iowa	2,339,900	39,400	2,379,300	11,100	1,500	12,600	0.53	11,100	900	12,000	0.50	658	0.03
Kansas	2,137,000	36,600	2,173,600	12,600	1,900	14,600	0.67	12,600	1,400	14,000	0.64	577	0.03
Kentucky	3,331,500	59,200	3,390,700	38,600	3,900	42,500	1.25	38,600	5,300	44,000	1.30	(1,461)	(0.04)
Louisiana	3,445,000	73,500	3,518,500	44,900	5,100	50,000	1.42	44,900	7,900	52,700	1.50	(2,739)	(0.08)
Maine	1,058,600	10,800	1,069,400	6,500	500	7,000	0.65	6,500	400	6,800	0.64	124	0.01
Maryland	4,547,800	55,000	4,602,900	37,300	3,300	40,600	0.88	37,300	3,200	40,500	0.88	101	0.00
Massachusetts	5,283,400	63,100	5,346,600	35,800	2,300	38,100	0.71	35,800	1,900	37,600	0.70	440	0.01
Michigan	7,578,400	98,200	7,676,600	62,800	6,300	69,100	0.90	62,800	6,200	69,000	0.90	93	0.00
Minnesota	4,115,000	44,900	4,159,900	22,300	1,900	24,300	0.58	22,300	1,300	23,600	0.57	672	0.02
Mississippi	2,205,500	46,100	2,251,700	19,600	3,200	22,900	1.02	19,600	3,400	23,000	1.02	(169)	(0.01)
Missouri	4,575,700	85,100	4,660,800	35,200	5,100	40,300	0.86	35,200	4,600	39,800	0.85	458	0.01
Montana	787,100	11,000	798,100	6,800	700	7,400	0.93	6,800	700	7,500	0.93	(13)	(0.00)
Nebraska	1,391,400	21,400	1,412,800	6,900	1,000	7,900	0.56	6,900	600	7,500	0.53	405	0.03
Nevada	2,148,500	29,000	2,177,400	19,300	2,600	21,900	1.00	19,300	2,700	22,000	1.01	(157)	(0.01)
				Additional Populations Estimation: Primary Method				Additional Populations Estimation: Alternative Method				Comparison between methods	
	Population			RNA+				RNA+				Difference	

© 2018 Rosenberg ES et al. *JAMA Network Open.*

State	ACS 2012-2016[a]	Additional Populations	Total[b]	Among NHANES population[c]	In Additional Populations	Total[b]	%	Among NHANES population[c]	In Additional Populations	Total[b]	%	RNA+	%
New Hampshire	1,046,300	11,700	1,058,000	7,200	500	7,700	0.73	7,200	400	7,600	0.72	87	0.01
New Jersey	6,810,300	80,600	6,890,900	43,400	3,800	47,200	0.68	43,400	2,900	46,200	0.67	933	0.01
New Mexico	1,557,100	20,900	1,578,000	25,000	1,600	26,700	1.69	25,000	3,100	28,200	1.78	(1,485)	(0.09)
New York	15,260,100	188,400	15,448,400	107,100	8,900	116,000	0.75	107,100	7,400	114,500	0.74	1,485	0.01
North Carolina	7,545,400	94,800	7,640,100	60,200	6,200	66,400	0.87	60,200	5,800	66,000	0.86	332	0.00
North Dakota	559,100	9,200	568,300	2,200	400	2,600	0.45	2,200	200	2,400	0.42	194	0.03
Ohio	8,787,100	151,400	8,938,500	81,500	8,100	89,600	1.00	81,500	8,900	90,300	1.01	(759)	(0.01)
Oklahoma	2,862,800	59,900	2,922,700	48,900	4,400	53,300	1.82	48,900	8,800	57,800	1.98	(4,460)	(0.15)
Oregon	3,086,200	34,800	3,120,900	45,700	2,900	48,700	1.56	45,700	5,200	50,900	1.63	(2,216)	(0.07)
Pennsylvania	9,888,700	166,900	10,055,600	84,500	9,300	93,900	0.93	84,500	9,500	94,000	0.94	(183)	(0.00)
Rhode Island	829,900	11,500	841,300	9,600	400	10,000	1.19	9,600	500	10,200	1.21	(152)	(0.02)
South Carolina	3,689,100	51,200	3,740,300	31,900	3,700	35,600	0.95	31,900	3,800	35,700	0.95	(100)	(0.00)
South Dakota	628,400	12,600	641,000	3,000	700	3,700	0.57	3,000	400	3,400	0.53	279	0.04
Tennessee	4,972,200	81,600	5,053,700	63,500	5,600	69,100	1.37	63,500	8,500	72,000	1.42	(2,883)	(0.06)
Texas	19,455,200	322,100	19,777,300	178,000	24,500	202,500	1.02	178,000	26,600	204,500	1.03	(2,036)	(0.01)
Utah	2,024,600	17,500	2,042,200	11,000	1,300	12,300	0.60	11,000	800	11,800	0.58	473	0.02
Vermont	499,100	4,700	503,800	3,500	200	3,700	0.73	3,500	200	3,700	0.73	34	0.01
Virginia	6,348,500	87,900	6,436,400	33,500	6,400	39,900	0.62	33,500	4,000	37,500	0.58	2,379	0.04
Washington	5,412,700	56,200	5,468,900	50,000	4,200	54,200	0.99	50,000	4,600	54,600	1.00	(391)	(0.01)
West Virginia	1,439,300	20,100	1,459,400	19,500	1,100	20,600	1.41	19,500	1,800	21,300	1.46	(692)	(0.05)
Wisconsin	4,384,900	64,700	4,449,600	24,000	3,900	27,900	0.63	24,000	2,600	26,600	0.60	1,371	0.03
Wyoming	437,600	6,700	444,300	3,200	500	3,700	0.82	3,200	400	3,600	0.81	59	0.01
**Total[b,d]**	**241,152,600**	**3,529,000**	**244,681,600[e]**	**2,035,100**	**231,600**	**2,266,700**	**0.93**	**2,035,100**	**239,600**	**2,274,800**	**0.93**	**(8,043)**	**0.00**

[a] Population sizes are estimated as of December 2016 based on American Community Survey 5-year estimates from 2012-2016 and include noninstitutionalized adults eligible for NHANES. This estimate includes 1,288,600 active-duty military personnel ineligible for NHANES, which cannot be removed at the state-level because population sizes are unavailable by home state of personnel.

[b] Values may not sum to total due to rounding.

[c] Number of infected persons is calculated by multiplying the prevalence percentage estimate by the adult population size, before rounding for presentation.

[d] Results are based on a regression model that incorporates data for the time period 1999-2016 and generates estimates via simulations. Accordingly, these results do not precisely sum to previous national totals for the 2013-2016 period.[26]

[e] Does not sum to previous 2013-2016 US total[26] due to the exclusion of persons incarcerated in federal prisons that are not assigned to state-specific populations.

Abbreviations: ACS, American Community Survey; NHANES, National Health and Nutrition Examination Survey

© 2018 Rosenberg ES et al. *JAMA Network Open.*

eReferences

1. National Center for Heather Statistics. NHANES Response Rates and Population Totals. https://wwwn.cdc.gov/nchs/nhanes/ResponseRates.aspx. Accessed October 26, 2018.
2. Zipf G, Chiappa M, Porter KS, Ostchega Y, Lewis BG, Dostal J. National health and nutrition examination survey: plan and operations, 1999-2010. *Vital and health statistics Ser 1, Programs and collection procedures.* 2013(56):1-37.
3. Centers for Disease Control and Prevention. Surveillance for Viral Hepatitis - United States, 2016. 2017; https://www.cdc.gov/hepatitis/statistics/2016surveillance/commentary.htm. Accessed January 1, 2018.
4. Trinidad JP, Warner M, Bastian BA, Minino AM, Hedegaard H. Using Literal Text From the Death Certificate to Enhance Mortality Statistics: Characterizing Drug Involvement in Deaths. *Natl Vital Stat Rep.* 2016;65(9):1-15.
5. Hedegaard H, Warner M, Minino AM. Drug Overdose Deaths in the United States, 1999-2016. *NCHS Data Brief.* 2017(294):1-8.
6. Katz J. Drug Deaths in America Are Rising Faster Than Ever. *The New York Times*2017.
7. Park H, Bloch M. How the Epidemic of Drug Overdose Deaths Rippled Across America. *The New York Times*2016.
8. Hurstak E, Rowe C, Turner C, et al. Using medical examiner case narratives to improve opioid overdose surveillance. *Int J Drug Policy.* 2018;54:35-42.
9. O'Donnell JK, Gladden RM, Seth P. Trends in Deaths Involving Heroin and Synthetic Opioids Excluding Methadone, and Law Enforcement Drug Product Reports, by Census Region - United States, 2006-2015. *MMWR Morb Mortal Wkly Rep.* 2017;66(34):897-903.
10. Rockett IRH, Caine ED, Connery HS, et al. Discerning suicide in drug intoxication deaths: Paucity and primacy of suicide notes and psychiatric history. *PLoS One.* 2018;13(1):e0190200.
11. Rockett IR, Hobbs G, De Leo D, et al. Suicide and unintentional poisoning mortality trends in the United States, 1987-2006: two unrelated phenomena? *BMC Public Health.* 2010;10:705.
12. Ruhm CJ. Geographic Variation in Opioid and Heroin Involved Drug Poisoning Mortality Rates. *Am J Prev Med.* 2017;53(6):745-753.
13. Slavova S, O'Brien DB, Creppage K, et al. Drug Overdose Deaths: Let's Get Specific. *Public Health Rep.* 2015;130(4):339-342.
14. Warner M, Paulozzi LJ, Nolte KB, Davis GG, Nelson LS. State Variation in Certifying Manner of Death and Drugs Involved in Drug Intoxication Deaths. *Acad Forensic Pathol.* 2013;3(2):231-237.
15. Buchanich JM, Balmert LC, Williams KE, Burke DS. The Effect of Incomplete Death Certificates on Estimates of Unintentional Opioid-Related Overdose Deaths in the United States, 1999-2015. *Public Health Rep.* 2018;133(4):423-431.
16. Rudd RA, Seth P, David F, Scholl L. Increases in Drug and Opioid-Involved Overdose Deaths - United States, 2010-2015. *MMWR Morb Mortal Wkly Rep.* 2016;65(5051):1445-1452.

© 2018 Rosenberg ES et al. *JAMA Network Open.*

17. Centers for Disease Control and Prevention. Provisional Counts of Drug Overdose Deaths, as of 8/6/2017. 2017; https://www.cdc.gov/nchs/data/health_policy/monthly-drug-overdose-death-estimates.pdf. Accessed October 1, 2017.

18. Warner M, Trinidad JP, Bastian BA, Minino AM, Hedegaard H. Drugs Most Frequently Involved in Drug Overdose Deaths: United States, 2010-2014. *Natl Vital Stat Rep.* 2016;65(10):1-15.

19. Novak SP, Kral AH. Comparing injection and non-injection routes of administration for heroin, methamphetamine, and cocaine users in the United States. *J Addict Dis.* 2011;30(3):248-257.

20. United States Department of Justice DEA. 2016 National Drug Threat Assessment: Summary. 2016.

21. Unick G, Rosenblum D, Mars S, Ciccarone D. The relationship between US heroin market dynamics and heroin-related overdose, 1992-2008. *Addiction.* 2014;109(11):1889-1898.

22. Seth P, Scholl L, Rudd RA, Bacon S. Overdose Deaths Involving Opioids, Cocaine, and Psychostimulants - United States, 2015-2016. *MMWR Morb Mortal Wkly Rep.* 2018;67(12):349-358.

23. Levy B, Spelke B, Paulozzi LJ, et al. Recognition and response to opioid overdose deaths-New Mexico, 2012. *Drug Alcohol Depend.* 2016;167:29-35.

24. Chirikov VV, Marx SE, Manthena SR, Strezewski JP, Saab S. Development of a Comprehensive Dataset of Hepatitis C Patients and Examination of Disease Epidemiology in the United States, 2013-2016. *Adv Ther.* 2018:1-16.

25. Moorman AC, Rupp LB, Gordon SC, et al. Long-Term Liver Disease, Treatment, and Mortality Outcomes Among 17,000 Persons Diagnosed with Chronic Hepatitis C Virus Infection: Current Chronic Hepatitis Cohort Study Status and Review of Findings. *Infect Dis Clin North Am.* 2018;32(2):253-268.

26. Hofmeister MG, Rosenthal EM, Barker LK, et al. Estimating prevalence of hepatitis C virus infection in the United States, 2013-2016. *Hepatology. In-press.*

27. United States Census Bureau. 2012-2016 ACS 5-year Estimates. 2018; https://www2.census.gov/programs-surveys/acs/summary_file/2016/data. Accessed February 1, 2018.

28. Kaeble D, Cowhig M. Correctional populations in the United States, 2016. 2018; https://www.bjs.gov/content/pub/pdf/cpus16.pdf. Accessed May 1, 2018.

29. United States Department of Housing and Urban Development. PIT and HIC Data Since 2007. 2017; https://www.hudexchange.info/resource/3031/pit-and-hic-data-since-2007. Accessed Febuary 1, 2018.

30. Harris-Kojetin L, Sengupta M, Park-Lee E, et al. Long-Term Care Providers and services users in the United States: data from the National Study of Long-Term Care Providers, 2013-2014. *Vital Health Stat 3.* 2016(38):x-xii; 1-105.

31. Akiyama MJ, Kaba F, Rosner Z, Alper H, Holzman RS, MacDonald R. Hepatitis C Screening of the "Birth Cohort" (Born 1945-1965) and Younger Inmates of New York City Jails. *Am J Public Health.* 2016;106(7):1276-1277.

© 2018 Rosenberg ES et al. *JAMA Network Open.*

32. Cocoros N, Nettle E, Church D, et al. Screening for Hepatitis C as a Prevention Enhancement (SHAPE) for HIV: an integration pilot initiative in a Massachusetts County correctional facility. *Public Health Rep.* 2014;129 Suppl 1:5-11.

33. de la Flor C, Porsa E, Nijhawan AE. Opt-out HIV and Hepatitis C Testing at the Dallas County Jail: Uptake, Prevalence, and Demographic Characteristics of Testers. *Public Health Rep.* 2017;132(6):617-621.

34. Kuncio DE, Newbern EC, Fernandez-Vina MH, Herdman B, Johnson CC, Viner KM. Comparison of risk-based hepatitis C screening and the true seroprevalence in an urban prison system. *J Urban Health.* 2015;92(2):379-386.

35. Mahowald MK, Larney S, Zaller ND, et al. Characterizing the Burden of Hepatitis C Infection Among Entrants to Pennsylvania State Prisons, 2004 to 2012. *J Correct Health Care.* 2016;22(1):41-45.

36. Schoenbachler BT, Smith BD, Sena AC, et al. Hepatitis C Virus Testing and Linkage to Care in North Carolina and South Carolina Jails, 2012-2014. *Public Health Rep.* 2016;131 Suppl 2:98-104.

37. Stockman LJ, Greer J, Holzmacher R, et al. Performance of Risk-Based and Birth-Cohort Strategies for Identifying Hepatitis C Virus Infection Among People Entering Prison, Wisconsin, 2014. *Public Health Rep.* 2016;131(4):544-551.

38. Coyle C, Viner K, Hughes E, et al. Identification and Linkage to Care of HCV-Infected Persons in Five Health Centers - Philadelphia, Pennsylvania, 2012-2014. *MMWR Morb Mortal Wkly Rep.* 2015;64(17):459-463.
